# TRPML1 loss disrupts lysosome–mitochondria homeostasis and sensitizes MDA-MB-231 triple-negative breast cancer cells to chemotherapy

**DOI:** 10.1016/j.jbc.2026.113596

**Published:** 2026-09-23

**Authors:** Alia K.R. Syeda, Shekoufeh Almasi, Cory J. Benson, Barry E. Kennedy, Ryan M. Yoast, Scott M. Emrich, Amari Delima-Baril, Logan Slade, Shanmugasundaram Pakkiriswami, Vishnu V. Vijayan, Unnikrishnan B. Sheela, Shashi Gujar, Thomas Pulinilkunnil, Mohamed Trebak, Yassine El Hiani

**Affiliations:** 1 Department of Physiology and Biophysics, Faculty of Medicine, Dalhousie University, Halifax, Nova Scotia, Canada; 2 Graduate Program in Cellular and Molecular Physiology, The Pennsylvania State University College of Medicine, Hershey, Pennsylvania, USA; 3 Department of Pharmacology and Chemical Biology, University of Pittsburgh School of Medicine, Pittsburgh, Pennsylvania, USA; 4 Vascular Medicine Institute, University of Pittsburgh School of Medicine, Pittsburgh, Pennsylvania, USA; 5 UPMC Hillman Cancer Center, University of Pittsburgh School of Medicine, Pittsburgh, Pennsylvania, USA; 6 Department of Microbiology and Immunology, Faculty of Medicine, Dalhousie University, Halifax, Nova Scotia, Canada; 7 Department of Biochemistry and Molecular Biology, Dalhousie University, Dalhousie Medicine New Brunswick, Saint John, New Brunswick, Canada

**Keywords:** TRPML1, lysosome-mitochondria crosstalk, Mitophagy, autophagic flux, lysosomal acidification, mitochondrial dynamics, chemoresistance

## Abstract

Signaling mechanisms at the lysosome–mitochondria interface form a critical network that enables cancer cells to maintain mitochondrial quality control, adapt to metabolic stress, and survive therapy. However, the incomplete understanding of the mechanisms coordinating this network has limited the development of effective therapies, especially for triple-negative breast cancers (TNBC). Here, we identify TRPML1 as an important regulator of lysosome-mitochondrial communication in MDA-MB-231 TNBC cells. We find that TRPML1 knockdown (ML1-KD) impaired mitochondrial respiration, oxidative substrate utilization, ATP production and redox balance in MDA-MB-231 cells, whereas comparable changes were not observed in non-cancerous MCF10A cells. ML1-KD reduced lysosomal acidification and impaired autophagic flux and was accompanied by reduced TFEB nuclear localization, impaired mitophagy, and alterations in mitochondrial maintenance proteins. These changes were accompanied by organellar proximity remodeling, with increased mitochondria–ER proximity and reduced mitochondria-lysosome proximity, together with altered cytosolic/mitochondrial Ca^2+^ responses, broad metabolic remodeling, G0/G1 arrest, and caspase-3/7-independent cell death. Importantly, ML1-KD cells showed enhanced responses to otherwise subeffective concentrations of doxorubicin and paclitaxel. Together, our findings support TRPML1-dependent lysosomal signaling as an important contributor to mitochondrial-metabolic resilience and chemotherapy responsiveness in MDA-MB-231 TNBC cells.

Cancer is characterized by uncontrolled cell growth and proliferation driven, at least in part, by perturbations in mitochondrial and lysosomal functions, two organelles central to tumor growth and survival (1, 2). Mitochondria are central to cellular bioenergetics, supporting biomass production and coordinating multiple signaling pathways, including calcium (Ca^2+^) homeostasis, reactive oxygen species (ROS) balance, cell cycle regulation, and apoptosis (3, 4, 5). Mitochondria-derived TCA cycle metabolites also regulate chromatin modifications, linking metabolic activity with gene expression in cancer cells (6, 7). Lysosomes, in turn, serve as signaling and metabolic hubs that support cancer progression (8, 9). Beyond their canonical function in macromolecule degradation and recycling, lysosomes enable cancer cells to cope with nutrient deprivation and stress conditions by regulating key signaling pathways, including mammalian target of rapamycin (mTOR), AMP-activated protein kinase (AMPK), and transcription factor EB (TFEB) (10, 11, 12, 13). Importantly, the functions of mitochondria and lysosomes extend beyond their respective organellar boundaries, allowing each organelle to influence the function and fate of the other. Accordingly, accumulating evidence highlights a functional interplay between mitochondria and lysosomes in maintaining cellular homeostasis and adapting to metabolic stress (14, 15). For example, the master transcriptional regulator of lysosomal function, TFEB, has been shown to regulate mitophagy (16, 17), cell death, and ATP production (18, 19). Disruption of the lysosomal pH modulator V-ATPase was associated with diminished mitochondrial respiration and increased cell death (20). Lysosomal Ca^2+^ release has also been shown to influence mitochondrial Ca^2+^ homeostasis (21), thereby impacting critical mitochondrial functions such as TCA cycle enzymatic activity and oxidative phosphorylation (22). Conversely, mitochondrial dysfunction can directly compromise lysosomal activity. For instance, deletion of mitochondrial dynamics proteins (*e.g.*, mitofusins, dynamin-related protein 1) or chemical inhibition of the electron transport chain (ETC) impairs lysosomal activity, leading to the formation of large lysosomal vacuoles (23, 24). These bidirectional relationships support a functional interdependence between these two organelles, whereby the integrity of one organelle is closely tied to the other, and dysfunction in either compromises cellular homeostasis (25, 26). While this dynamic dependency supports cellular resilience, it may also create a metabolic vulnerability for cancer cells, which must balance rapid growth demands with nutrient availability and energy generation. Defining the molecular mechanisms that coordinate lysosome–mitochondria communication may therefore uncover previously unrecognized vulnerabilities in cancer.

Recently, we (27) and others (28) have demonstrated the important role of Transient Receptor Potential Mucolipin 1 (TRPML1), a lysosomal Ca^2+^-release channel, in promoting breast cancer survival and proliferation *in vitro* and in xenograft models. Specifically, we showed that downregulation of TRPML1 impairs triple-negative breast cancer (TNBC) growth and invasion by inhibiting mTORC1 activity and facilitating the release of lysosomal ATP into the extracellular space, respectively. Here, we identify TRPML1 as an important regulator of lysosome–mitochondria communication in MDA-MB-231 TNBC cells. Our findings reveal that TRPML1 downregulation alters mitochondrial structure and bioenergetics, disrupts mitophagy and autophagy flux, and remodels organelle organization, reducing mitochondria-lysosome proximity while increasing mitochondria-ER proximity. These changes are accompanied by metabolic stress, cell cycle arrest, caspase-3/7-independent cell death, and enhanced chemosensitivity. Consistent with these cellular phenotypes, complementary analysis using the public OncoGenomics database [GSE22133−GPL5345 (29) and GSE-42568 (30)] revealed an association between lower TRPML1 mRNA expression and improved survival in breast cancer cohorts. Collectively, these data support TRPML1-dependent lysosomal signaling as a potential vulnerability in MDA-MB-231 TNBC cells and reveal a signaling profile distinct from the related mucolipin TRPML3 (31), which operates within a different lysosomal pH-dependent signaling context (31, 32).

## Results

### TRPML1 knockdown suppresses clonogenic growth and promotes G0/G1 cell cycle arrest and caspase-3/7-independent cell death in MDA-MB-231 cells

Building on our previous work demonstrating that TRPML1 is upregulated in multiple TNBC cells models and supports TNBC growth, we first confirmed TRPML1 expression and knockdown in the cellular models used in the present study. TRPML1 mRNA expression was significantly higher in MDA-MB-231 than in non-tumorigenic MCF10A cells (Fig. 1*A*), and efficient TRPML1 knockdown (ML1-KD) was confirmed by RT-qPCR in both cell models (Fig. 1*B*). ML1-KD significantly reduced colony formation in MDA-MB-231 cells compared with PLKO control cells, whereas no detectable effect was observed in MCF10A cells (Fig. 1*C*). Consistent with our previous findings in additional TNBC models, ML1-KD also reduced proliferation in HCC1806 and HS578T cells (Fig. S1). To further investigate the cellular basis growth phenotype in MDA-MB-231 cells, we examined cell cycle distribution and cell death markers. ML1-KD increased the proportion of cells in G0/G1, with corresponding reductions in S and G2/M phases (Fig. 2*A*), consistent with G0/G1 cell-cycle arrest, and was accompanied by decreased levels of Cyclin B1 and Cyclin D1, increased expression of p27, and reduced levels of E2F1 (Fig. 2*B*), a critical transcription factor for cell cycle progression (33). ML1-KD also significantly increased the proportion of Annexin V-positive cells, consistent with increased cell death (Fig. 2*C*). Analysis of cell lysates revealed elevated levels of cytochrome C and cleaved caspase 9, but no detectable cleaved caspase 3 or 7, indicating that cell death occurred without detectable activation of the executioner caspase-3/7 (Fig. 2*D*). ML1-KD also decreased phosphorylated BCL2 (pBCL2(S70)) and increased endonuclease G (EndoG) levels, whereas apoptosis-inducing factor (AIF) remained unchanged (Fig. 2*E*). Notably, the examined cell cycle and cell death markers were not significantly altered by ML1-KD in the non-cancerous MCF10A cells (Fig. 2*F*). Because lysosomal membrane permeabilization (LMP) with cathepsin release can contribute to caspase-independent cell death, we assessed lysosomal integrity. Confocal microscopy analysis revealed increased accumulation and colocalization of Cathepsin B within LAMP1-positive lysosomes in ML1-KD1 and ML1-KD3 cells compared to PLKO controls (Fig. S2), consistent with lysosomal retention rather than cytosolic redistribution. These observations provided no evidence for overt LMP as a major contributor to cell death under these conditions. Overall, these results indicate that TRPML1 knockdown reduces MDA-MB-231 cell growth through G0/G1 cycle-cycle arrest and caspase-3/7-independent cell death, with no evidence for overt LPM as a major contributor to cell death under these conditions (34).

**Figure 1:**
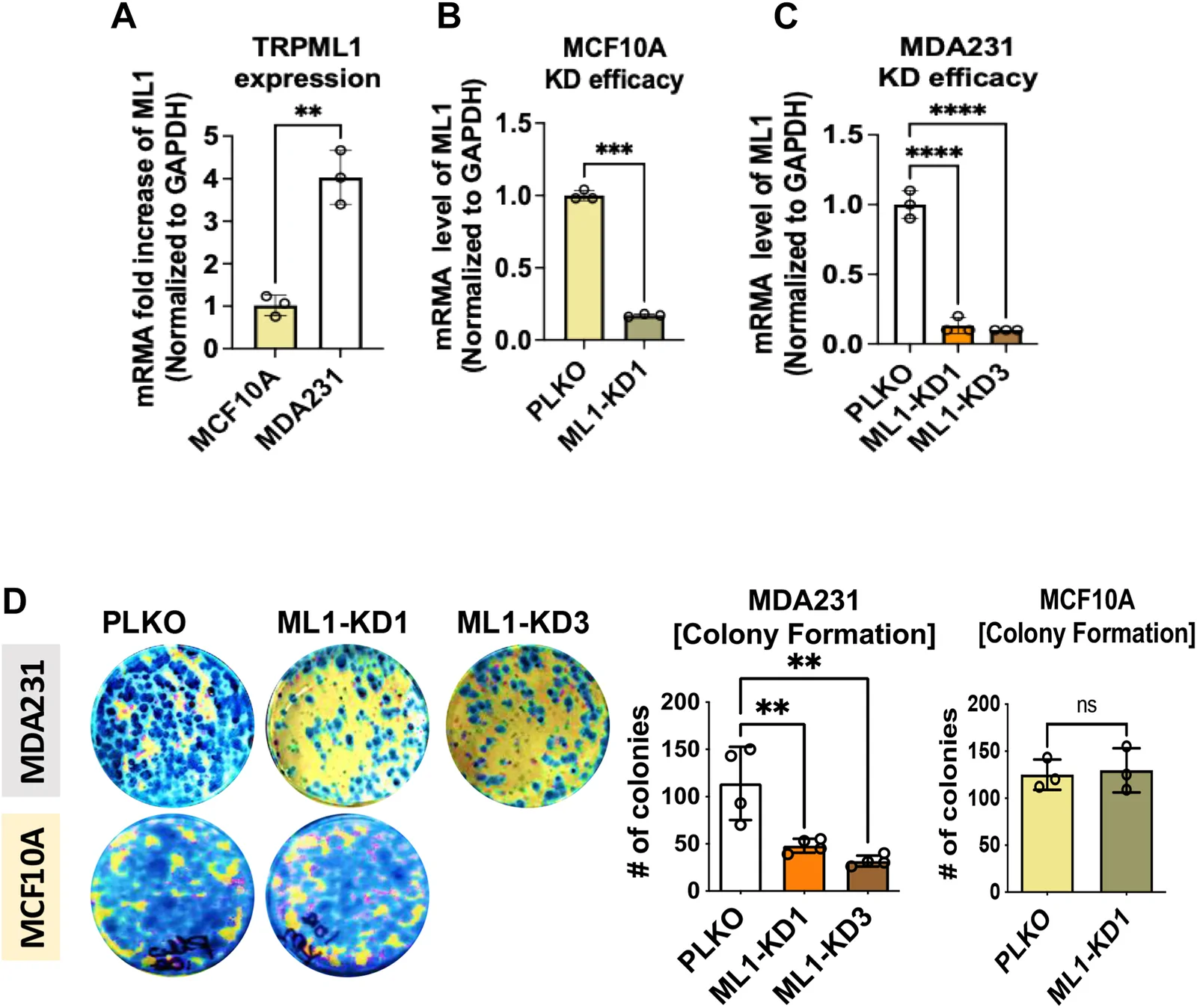
**TRPML1 knockdown reduces clonogenic growth in MDA-MB-231 cells but not MCF10A cells.***A*, TRPML1 mRNA expression in MDA-MB-231 and non-tumorigenic MCF10A cells measured by RT-qPCR. *B* and *C*, RT-qPCR validation of TRPML1 knockdown in MDA-MB-231 cells expression using two independent TRPML1-targeting ShRNAs (ML1-KD1 and ML1-KD3) and in MCF10A cells expressing ML1-KD1, relative to their respective PLKO controls. *D*, representative images and quantification of colony formation in PLKO and ML1-KD in MDA-MB-231 and MCF10A cells. Data are presented as mean ± SD, from three independent experiments. Statistical significance was determined by one-way ANOVA with Tukey’s multiple comparisons test for PLKO *versus* ML1-KD1/Ml1-KD3 of MDA-MB-231, and unpaired *t* test and Mann–Whitney *U* test for PLKO and ML1-KD1 of MCF10A cells. ∗∗*p* < 0.01; and ∗∗∗*p* < 0.001; ns, not significant.

**Figure 2:**
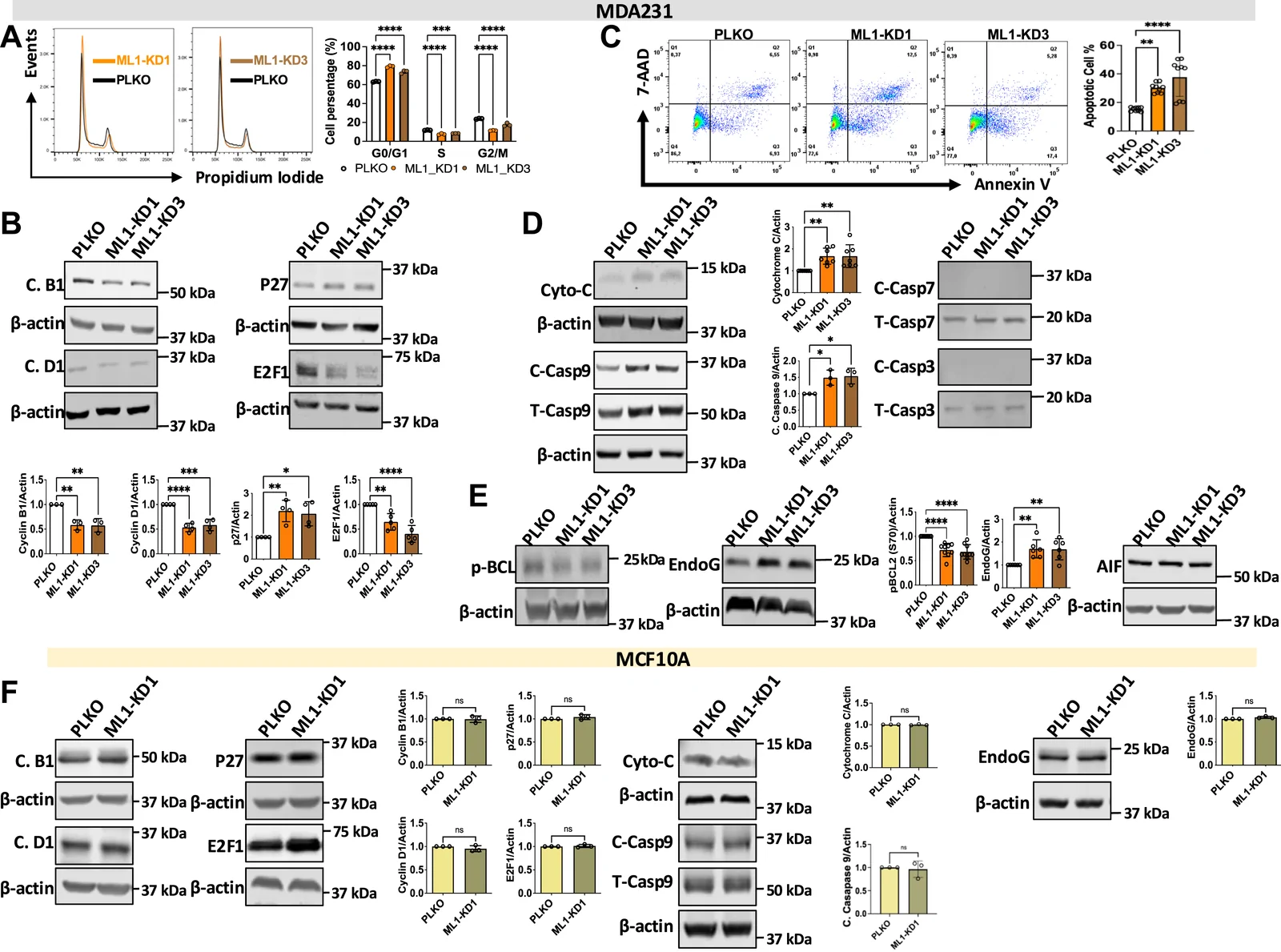
**TRPML1 knockdown promotes G0/G1 cell cycle arrest and caspase-3/7-independent cell death in MDA-MB-231 cells.***A*, representative propidium iodide (PI) flow-cytometry profiles of cell cycle distribution in PLKO *versus* ML1-KD MDA-MB-231 cells. Bar graph shows the mean ± SD percentage of cells in G0/G1, S, and G2/M phases from a representative data out of three independent experiments. *B*, Representative Western blots and corresponding quantification showing mean ± SD of Cyclin B1 (n = 3), Cyclin D1 (n = 4), p27 (n = 4), and E2F1 (n = 5) in PLKO and ML1-KD MDA-MB-231 cells. *C*, Representative Annexin V/7-AAD flow-cytometry plots in PLKO and ML1-KD MDA-MB-231 cells. Bar graph shows mean ± SD percentages of apoptotic cells, from three independent experiments; *D*, Representative Western blots of cell-death-associated proteins with corresponding quantification of Cytochrome C (n = 7) and Cleaved caspase 9 (n = 3). *E*, p-BCL2(S70) (n = 8), EndoG (n = 6) and AIF in PLKO and ML1-KD MDA-MB-231 cells. *F*, representative Western blots and corresponding quantification Cyclin B1, Cyclin D1, p27, E2F1, cytochrome c, cleaved-caspase 9, and EndoG in PLKO and ML1-KD1 MCF10A cells, from three independent experiments (n = 3). Western blot quantifications were normalized to β-Actin and expressed relative to the corresponding PLKO control. The β-Actin control of Cyclin D1 (Fig. 2*F*) is the same as the β-Actin control used for MFN2 (Fig. 4*E*) since the same membrane was used to prob for both proteins. Data are presented as mean ± SD; n represents independent biological experiments; Statistical significance was determined by one-way ANOVA with Tukey’s multiple comparisons test for PLKO *versus* ML1-KD1/Ml1-KD3 MDA-MB-231, and unpaired *t* test and Mann-Whitney U test for PLKO and ML1-KD1 MCF10A cells. ∗*p* < 0.05, ∗∗*p* < 0.01, and ∗∗∗*p* < 0.001. ns, not significant.

### TRPML1 knockdown impairs mitochondrial respiration and cellular bioenergetics in MDA-MB-231 cells

To determine whether the reduced growth and cell-death phenotype observed in ML1-KD cells were associated with mitochondrial dysfunction, we performed the Seahorse XF Mito Stress Test in MDA-MB-231 and MCF10A cells (Fig. 3, *A* and *B*). Oxygen consumption rate (OCR) and extracellular acidification rate (ECAR) were monitored following sequential mitochondrial perturbation, and OCR/ECAR ratios were assessed under basal, maximal, and ATP-linked respiratory conditions to evaluate the balance between oxidative phosphorylation and glycolytic flux. ML1-KD in MDA-MB-231 cells led to a marked decrease in both basal and maximal OCR/ECAR ratios, as well as in the ATP-linked OCR/ECAR parameter, consistent with impaired mitochondrial bioenergetics (Fig. 3, *A* and *B*). In contrast, ML1-KD had no effect on OCR/ECAR ratios in MCF10A cells (Fig. 3, *A* and *B*). To determine whether this bioenergetic defect reflected impaired mitochondrial respiratory capacity, we next performed high-resolution oxygraphy in MDA-MB-231 cells. ML1-KD cells exhibited reduced ADP-stimulated respiration supported by pyruvate and malate, and this defect remained evident after succinate addition, compared to PLKO control cells, consistent with impaired oxidative phosphorylation (Fig. 3*C*). We next assessed substrate dependence using CENCAT, a flow cytometry-based assay of cellular metabolic dependence on oxidative substrates (Fig. 3*D*) (35). ML1-KD MDA-MB-231 cells displayed reduced translational output (Fig. 3*E*). Analysis of the CENCAT profiles further revealed reduced mitochondrial dependence and decreased fatty acid oxidation (FAO) and amino acid oxidation (AAO) (Fig. 3, *F* and *G*) capacity, consistent with reduced oxidative substrate utilization. To determine whether these defects translated into impaired energy homeostasis, we directly measured total cellular ATP levels. Cellular ATP levels were significantly reduced in ML1-KD MDA-MB-231 cells compared to PLKO controls, while ATP content in MCF10A cells remained unchanged (Fig. 3*H*). We next examined electron transport chain (ETC) protein abundance. Western blot analysis showed that ML1-KD decreased levels of complexes I, II, and IV (Fig. 3*I*). ML1-KD was also accompanied by increased levels of both total and mitochondrial ROS (Fig. 3*K*), indicating redox imbalance. Notably, MCF10A cells showed no significant changes in ETC levels (Fig. 3*I*) or ROS production (Fig. 3*K*) upon ML1-KD. Together, these findings indicate that knockdown of TRPML1 impairs mitochondrial bioenergetics in MDA-MB-231 cells, characterized by reduced respiratory capacity and oxidative substrate utilization, decreased cellular ATP, altered ETC protein levels, and increased ROS, whereas these changes were not observed in MCF10A cells.

**Figure 3:**
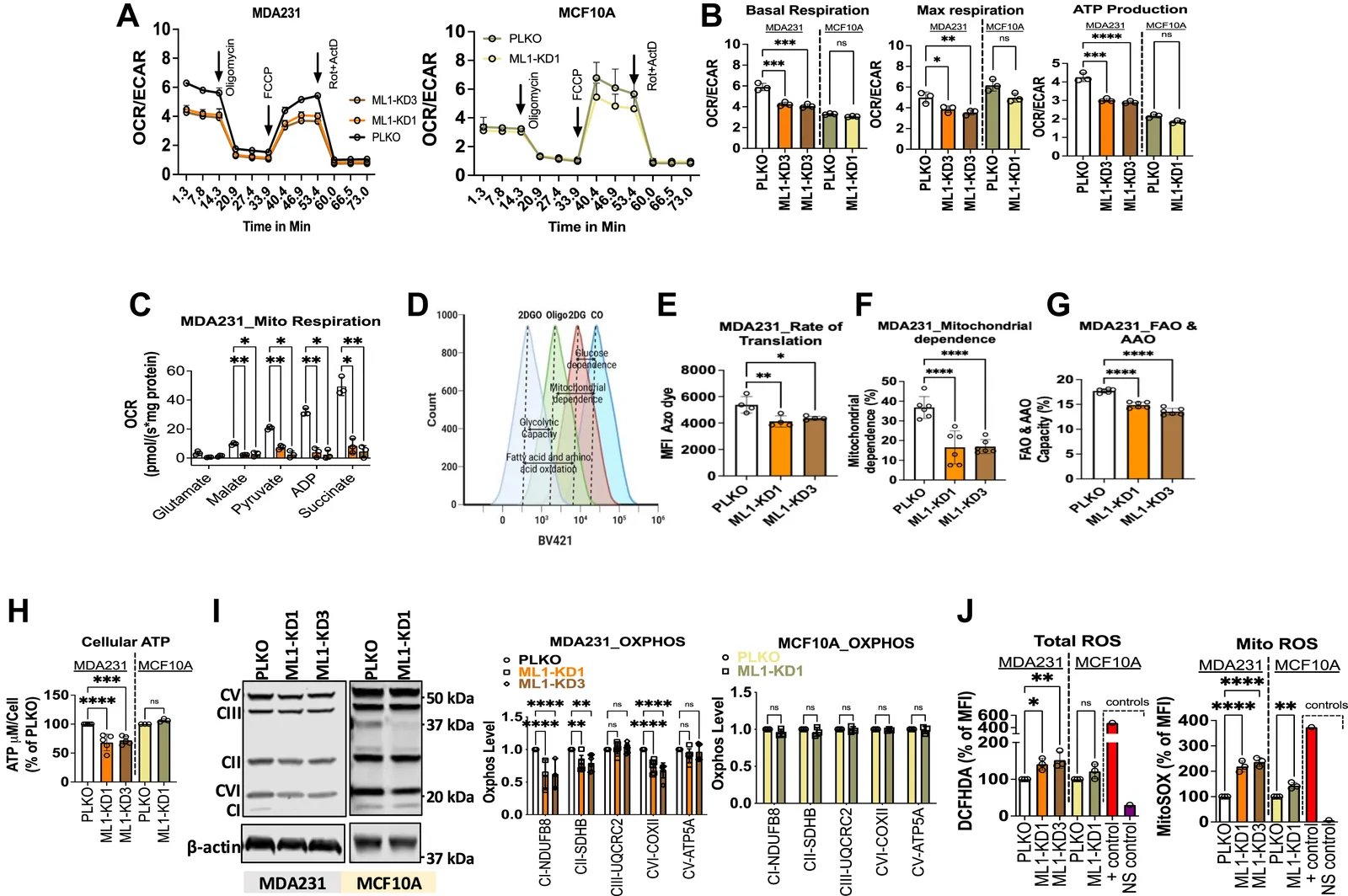
**TRPML1 knockdown impairs mitochondrial respiration and cellular bioenergetics in MDA-MB-231 TNBC cells.***A*, OCR/ECAR ratio assessed by Seahorse XF Mito Stress Test in PLKO and ML1-KD MDA-MB-231 and MCF10A cells. *B*, quantification of Basal, maximal, and ATP-linked OCR/ECAR ratios in PLKO and ML1-KD MDA-MB-231 and MCF10A cells. *C*, high-resolution mitochondrial respiration measured in PLKO and ML1-KD of MDA-MB-231 cells using Oroboros respirometry following sequential addition of glutamate, Malate, Pyruvate, ADP and Succinate. *D*, schematic of metabolic monitoring using CENCAT conditions; and the rate of translation. *E–G*, quantification of translational output, mitochondrial dependency, and FAO/AAO capacity calculated from the CENCAT measurements (see Experimental procedures). *H*, quantification of cellular ATP levels in PLKO and ML1-KD of MDA-MB-231 and MCF10A cells. *I*, representative Western blots and corresponding quantification of representative subunits of OXPHOS complexes I–V in PLKO and ML1-KD of MDA-MB-231 and MCF10A cells, with β-Actin serving as a loading control. *J*, quantification of total and mitochondrial ROS using DCFHDA and MitoSOX, respectively, in PLKO and TRPML1 KD of MDA-MB-231 and MCF10A cells. Data are presented as means ± SD from three independent experiments. Statistical significance was determined by one-way ANOVA with Tukey’s multiple comparison test for PLKO *versus* ML1-KD1/Ml1-KD3 MDA-MB-231, and unpaired *t* test and Mann-Whitney U test for PLKO and ML1-KD1 MCF10A cells. ∗*p* < 0.05, ∗∗*p* < 0.01, and ∗∗∗*p* < 0.001. ns, not significant.

### TRPML1 knockdown induces mitochondrial fragmentation and is associated with organelle proximity remodeling in MDA-MB-231 cells

Because mitochondrial function is closely coupled to network architecture, we next asked whether TRPML1 knockdown altered mitochondrial morphology. MitoTracker staining and confocal microscopy revealed that mitochondria in MDA-MB-231 PLKO control cells were distributed throughout the cytoplasm, whereas they appeared fragmented and more concentrated in the perinuclear region in ML1-KD MDA-MB-231 cells (Fig. 4*A*). Quantitative analysis of confocal images confirmed increased mitochondrial fragmentation and reduced mitochondrial volume in ML1-KD cells (Fig. 4*B*). In contrast, mitochondrial morphology and volume were unchanged in MCF10A cells (Fig. 4*B*). Analysis of mitochondrial fission markers revealed no significant changes in MFF abundance or DRP1 phosphorylation at Ser616 or Ser637 (Fig. 4*C*). However, ML1-KD MDA-MB-231 cells showed reduced levels of mitofusin 1 (MFN1), mitofusin 2 (MFN2), and optic atrophy 1 (OPA1) (Fig. 4*D*). These changes were not observed in MCF10A cells, in which MFN1, MFN2, and OPA1 levels remained unchanged (Fig. 4*E*). Thus, mitochondrial fragmentation following ML1-KD was associated with reduced abundance of mitochondrial fusion proteins, while the examined fission-associated markers remained unchanged. To determine whether this fragmentation was accompanied by structural and special remodeling, we next examined mitochondrial ultrastructure and inter-organelle proximity using TEM and 3D confocal microscopy. While we observed no differences in mitochondrial area between PLKO and ML1-KD of MDA-MB-231 cells (Fig. 5, *A* and *B*), ML1-KD MDA-MB-231 cells exhibited increased mitochondrial circularity and decreased aspect ratio (Fig. 5, *A*, *C* and *D*), consistent with the fragmented morphology observed by confocal microscopy. Mitochondria of ML1-KD MDA-MB-231 cells also displayed increased cristae density compared to PLKO control cells (Fig. 5*E*). TEM analysis additionally showed increased ER-mitochondria proximity, consistent with increased mitochondria-associated membranes (MAM) in ML1-KD cells (Fig. 5, *A* and *F*). Consistent with this ultrastructural observation, 3D Z-stack confocal microscopy showed a marked increase in the surface-to-surface proximity between MitoTracker-labeled mitochondria and Sec61-NEON-labeled ER (Fig. 5, *G* and *H*). Conversely, proximity between LysoTracker-labeled lysosomes and Mito-GFP-labeled mitochondria was reduced in ML1-KD cells (Fig. 5, *I* and *J*). Collectively, these findings indicate that TRPML1 knockdown is associated with mitochondrial fragmentation, reduced abundance of fusion-associated proteins, and spatial organelle remodeling characterized by increased mitochondria-ER proximity and reduced mitochondria–lysosome proximity in MDA-MB-231 cells.

**Figure 4:**
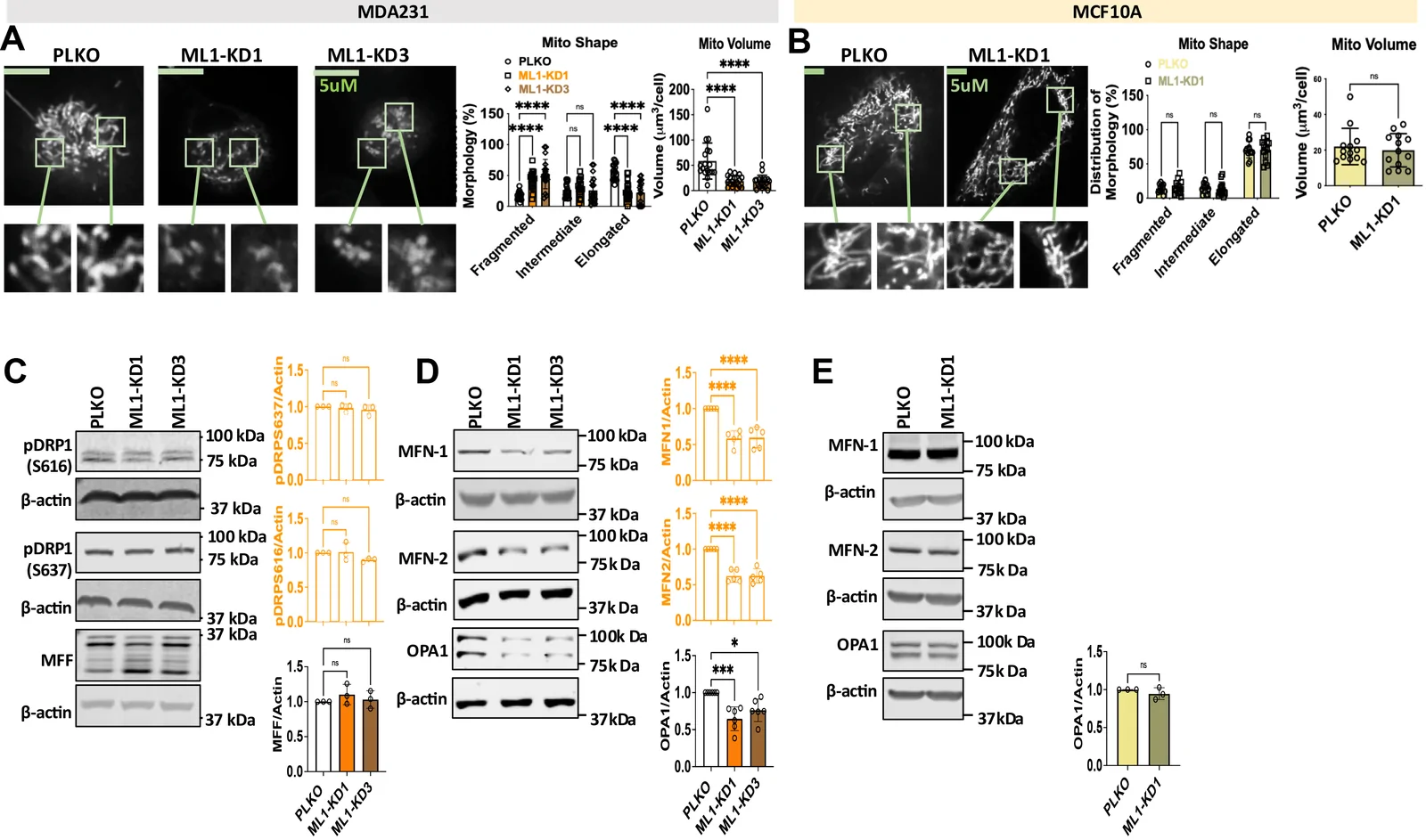
**TRPML1 knockdown induces mitochondrial fragmentation and is associated with reduced mitochondrial fusion protein abundance in MDA-MB-231 cells.***A*, representative images of mitochondria assessed by MitoTracker *Red* staining in PLKO and ML1-KD from MDA-MB-231 by live-cell confocal microscopy (100x; maximum-intensity projection shown in *grayscale*), with two magnified insets beneath each image highlighting mitochondrial morphology. Corresponding quantitative analysis of mitochondrial morphology and volume was performed using Imaris 10.2 software. Mitochondria were classified into 3 groups: fragmented, intermediate, and elongated. Data were obtained from three independent biological experiments, with 18 cells analyzed per condition; Scale bar, 5 μm. *B*, Representative *grayscale* images and corresponding quantification (with 13 cells analyzed per condition) of mitochondrial morphology and volume in PLKO and ML1-KD1 MCF10A cells, analyzed as described in (*A*). *C*, representative Western blots and corresponding quantification of the mitochondrial fission-associated proteins p-[S616]DRP1 (n = 3), p-[S637]DRP1 (n = 3), and MFF (n = 3) in PLKO and ML1-KD of MDA-MB-231 cells. *D*, representative Western blots and corresponding quantification of the mitochondrial fusion-associated proteins MFN1 (n = 5), MFN2 (n = 5) and OPA1 (n = 6) in PLKO and ML1-KD of MDA-MB-231 cells. *E*, representative Western blots and corresponding quantification of the mitochondrial fusion of MFN1 (n = 3), MFN2 (n = 3), and OPA1 (n = 3) in PLKO and ML1-KD1 MCF10A cells. Western blot quantifications were normalized to β-Actin and expressed relative to the corresponding PLKO control. The β-Actin control of MFN2 (Fig. 4*E*) is the same as the β-Actin control used for Cyclin D1 (Fig. 2*F*) since the same membrane was used to prob for both proteins. Data are presented as mean ± SD, with n representing independent experiments; Statistical significance was determined by one-way ANOVA with Tukey’s multiple comparison test for PLKO *versus* ML1-KD1/Ml1-KD3 MDA-MB-231, and unpaired *t* test and Mann-Whitney U test for PLKO and ML1-KD1 MCF10A cells. ∗*p* < 0.05, ∗∗*p* < 0.01, and ∗∗∗*p* < 0.001. ns, not significant.

**Figure 5:**
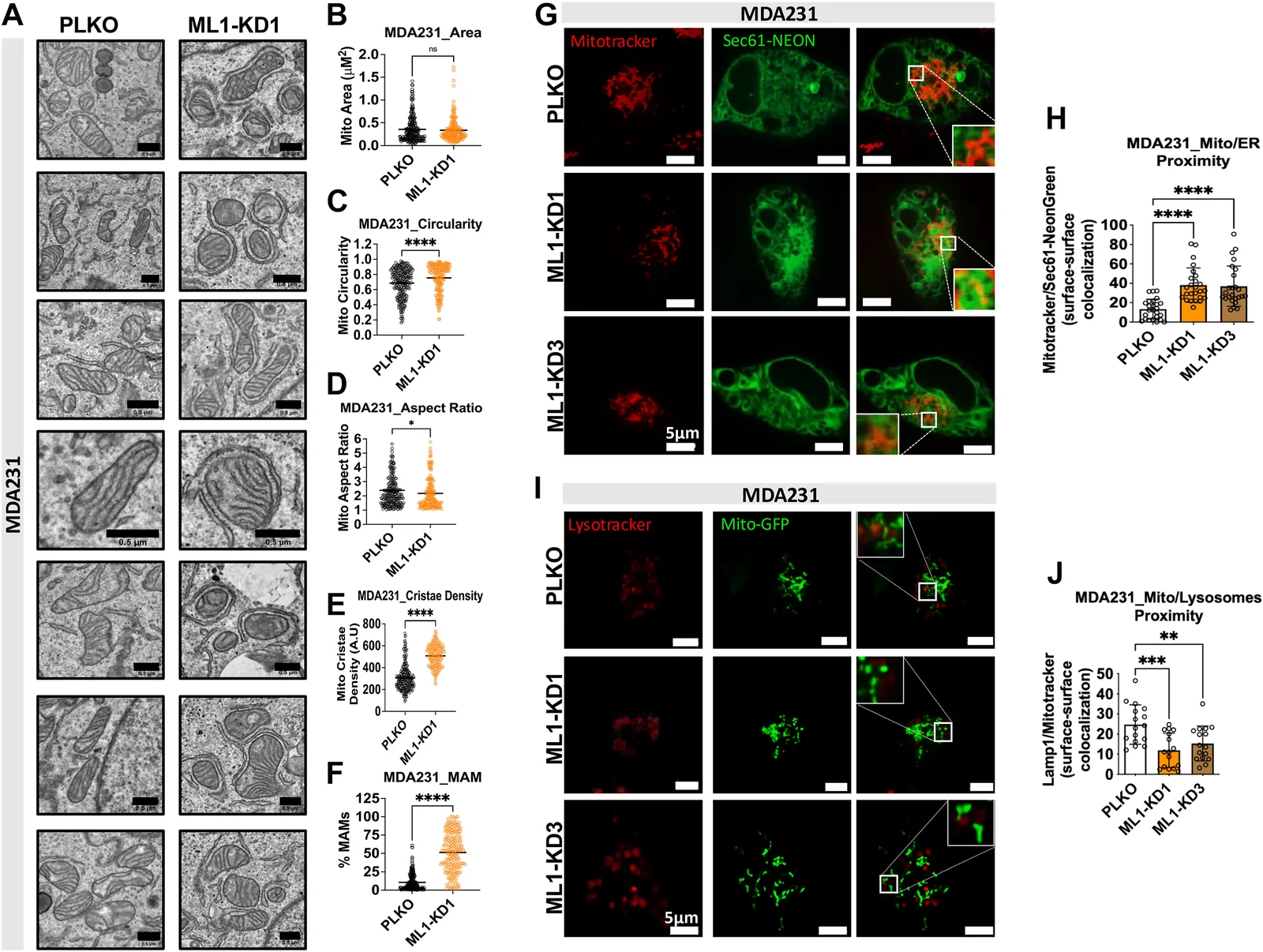
**TRPML1 knockdown alters mitochondrial ultrastructure and is associated with increased ER-mitochondria proximity and reduced mitochondria-lysosome proximity in MDA-MB-231 TNBC cells.***A*, representative TEM images of PLKO and ML1-KD1 MDA-MB-231 cells. Scale bar: 0.5 μm. *B–F*, quantification of mitochondria area (*B*), circularity (*C*), aspect ratio (*D*), cristae density (*E*), and MAMs (*F*) of PLKO and ML1-KD1 MDA-MB-231 cells using ImageJ. *G*, representative single Z-Stack planes acquired by live-cell microscopy (100x) from PLKO and ML1-KD MDA-MB-231 cells stained with MitoTracker (mitochondria, *red*) and Sec61-NEON (ER, *green*). *Merged panels* show the degree of overlap between mitochondrial and ER volumes. Scale bar: 0.5 μm. *H*, quantification of the % of surface-to-surface colocalization between mitochondrial and ER volumes in PLKO and ML1-KD MDA-MB-231 cells. Data are presented as mean ± SD and were obtained from three independent biological experiments, with 25 cells analyzed per condition. *I*, representative single Z-Stack planes acquired by live-cell microscopy (100x) from PLKO and ML1-KD MDA-MB-231 cells stained with Lysotracker (lysosome, *red*) and MitoTracker (mitochondria, *green*). *Merge panels* show the degree of overlapping between lysosomes and mitochondrial volumes. Scale bar: 5 μm. *J*, quantification of the % of surface-to-surface colocalization between lysosomal and mitochondrial volumes in PLKO and ML1-KD MDA-MB-231 cells; Data are presented as mean ± SD and were obtained from three independent biological experiments, with 16 cells analyzed per condition. Statistical significance was determined by unpaired *t* test and Mann–Whitney *U* test for *panels B–F* and one-way ANOVA with Tukey’s multiple comparison test for *panels H* and *J*. ∗*p* < 0.05, ∗∗*p* < 0.01, and ∗∗∗*p* < 0.001. ns, not significant.

### TRPML1 knockdown enhances signaling associated with autophagy initiation but impairs autophagic flux

The accumulation of fragmented mitochondria with reduced lysosomal proximity led us to ask whether ML1-KD impairs autophagic clearance. We first examined signaling associated with autophagy initiation through the mTORC1- and AMPK-ULK1 pathways (36, 37, 38). Consistent with our previous findings, phosphorylation of P70S6K at Thr389 was decreased in ML1-KD MDA-MB-231 cells compared to PLKO control cells (Fig. 6*A*), consistent with reduced mTORC1 signaling. ML1-KD lysates also displayed reduced phosphorylation of ULK1 at Ser757, an inhibitory mTORC1-dependent site on ULK1 (Fig. 6*A*). In parallel, ML1-KD increased AMPK phosphorylation at Thr172 (Fig. 6*B*) and increased phosphorylation of ULK1 at Ser555 (Fig. 6*B*), consistent with enhanced AMPK-ULK1 signaling associated with autophagy initiation (39, 40). These signaling changes were not observed in MCF10A cells (Fig. 6, *A* and *B*). To determine the effect of TRPML1 knockdown on autophagic flux, we analyzed LC3-II in the presence and absence of bafilomycin A1 (BafA) (41) (Fig. 6, *C–G*). Basal LC3-II levels were elevated in ML1-KD compared to PLKO control cells in BafA-untreated MDA-MB-231 cells (Fig. 6, *C* and *D*), whereas BafA produced little further LC3-II accumulation in ML1-KD cells compared to PLKO controls. Accordingly, calculated autophagic flux was reduced following ML1-KD (Fig. 6*E*), supporting impaired autophagosome clearance rather than increased autophagosome accumulation alone. Notably, TRPML1 knockdown did not affect LC3-II accumulation or calculated autophagic flux in MCF10A cells (Fig. 6, *C*, *F* and *G*). We next examined autophagosome maturation using the tandem pMRX-IP-RFP-GFP-LC3 (42, 43). In this assay, autophagosomes exhibit yellow puncta due to colocalization of GFP (green) with RFP (red). Following delivery to acidic autolysosomal compartments, GFP fluorescence is quenched while RFP fluorescence is retained, resulting in red-only puncta. MDA-MB-231 PLKO control cells showed predominantly red-only puncta, whereas ML1-KD MDA-MB-231 cells displayed an increased proportion of yellow puncta, consistent with impaired progression to acidic autolysosomal compartments (Fig. 7*A*). Because productive autophagic degradation depends on lysosomal acidification (44, 45, 46, 47), we next assessed lysosomal acidity using the complementary LysoTracker and LysoSensor probes whose accumulation in lysosomal compartments depends on an acidic luminal environment, and LysoSensor, whose fluorescence is pH-sensitive (31). ML1-KD MDA-MB-231 cells displayed reduced LysoTracker and LysoSensor signals compared with PLKO control cells, consistent with reduced lysosomal acidification (Fig. 7, *B* and *C*). Conversely, overexpression of TRPML1 in MDA-MB-231 PLKO cells increased LysoTracker signal, supporting a relationship between TRPML1 abundance and lysosomal acidity (Fig. 7*D*). Together, these findings indicate that ML1-KD creates a paradoxical autophagy phenotype in MDA-MB-231 cells: signaling associated with autophagy initiation is enhanced, with reduced mTORC1 signaling and increased AMPK-ULK1 signaling, while downstream autophagic flux is impaired in association with reduced lysosomal acidification and impaired progression to acidic autolysosomal compartments.

**Figure 6:**
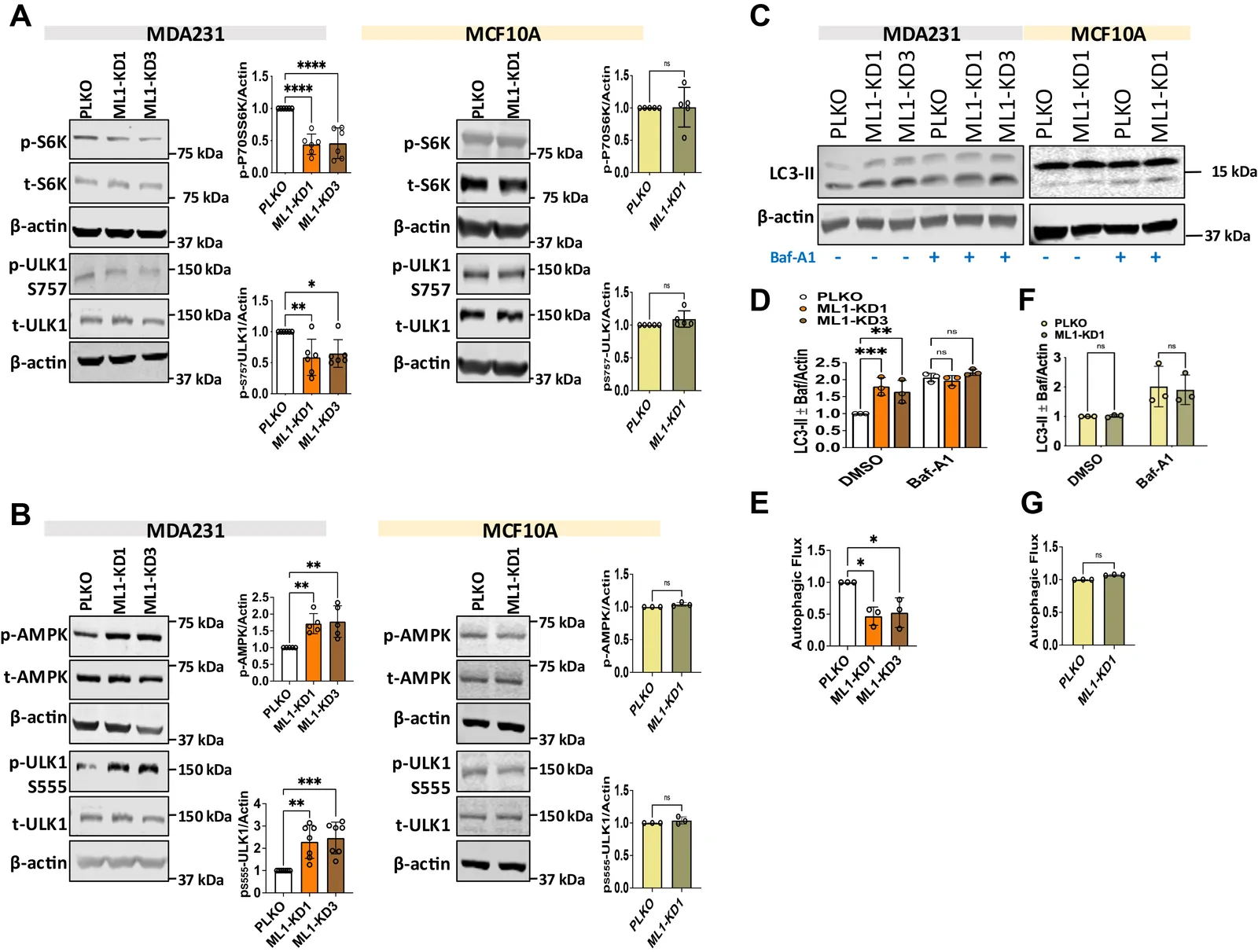
**TRPML1 knockdown enhances autophagy-initiation signaling but impairs autophagic flux in MDA-MB-231 TNBC cells.***A*, representative Western blot of phospho and total S6K and ULK1 in PLKO and ML1-KD MDA-MB-231 and MCF10A cells, with corresponding quantification of phospho S6K Thr389 and (n = 6) phosphor-ULK1 Ser757 (n = 6) in MDA-MB-231 cells, and S6K Thr389 and (n = 5) phosphor-ULK1 Ser757 (n = 5) in MCF10A cells. *B*, representative Western blots of phospho and AMPK and ULK1 Ser555, with corresponding quantification of phosphor-AMPK Thr172 (n = 5) and phospho-ULK1 Ser555 (n = 7) in MDA-MB-231 cells, and phosphor-AMPK Thr172 (n = 3) and phosphor-ULK1 Ser555 (n = 3) in MCF10A cells. *C*, representative Western blot of LC3-II in PLKO and ML1-KD MDA-MB-231 and MCF10A cells treated with DMSO or 200 nM bafilomycin A1 (BafA) for 2 h. *D*, Quantification of LC3 II levels in DMSO- and BafA-treated PLKO and ML1-KD in MDA-MB-231 (n = 3). *E*, calculated autophagic flux in MDA-MB-231 cells, defined for each condition as (LC3II/β-Actin)_BafA_–minus (LC3II/β-Actin)_DMSO_ and expressed relative to PLKO (n = 3). *F*, quantification in DMSO- and BafA-treated PLKO and ML-KD1 MCF10A cells (n = 3). *G*, calculated autophagic flux in MCF10A cells, determined as described in (*E*) and expressed relative to the PLKO (n = 3). Western blot quantifications were normalized to β-Actin and expressed relative to the corresponding PLKO. Data are presented as mean ± SD, with n representing independent experiments. Statistical significance was determined by one-way ANOVA with Tukey’s multiple comparison test for PLKO *versus* ML1-KD1/Ml1-KD3 MDA-MB-231, and unpaired *t* test and Mann–Whitney *U* test for PLKO and ML1-KD1 MCF10A cells. ∗*p* < 0.05, ∗∗*p* < 0.01, and ∗∗∗*p* < 0.001. ns, not significant.

**Figure 7:**
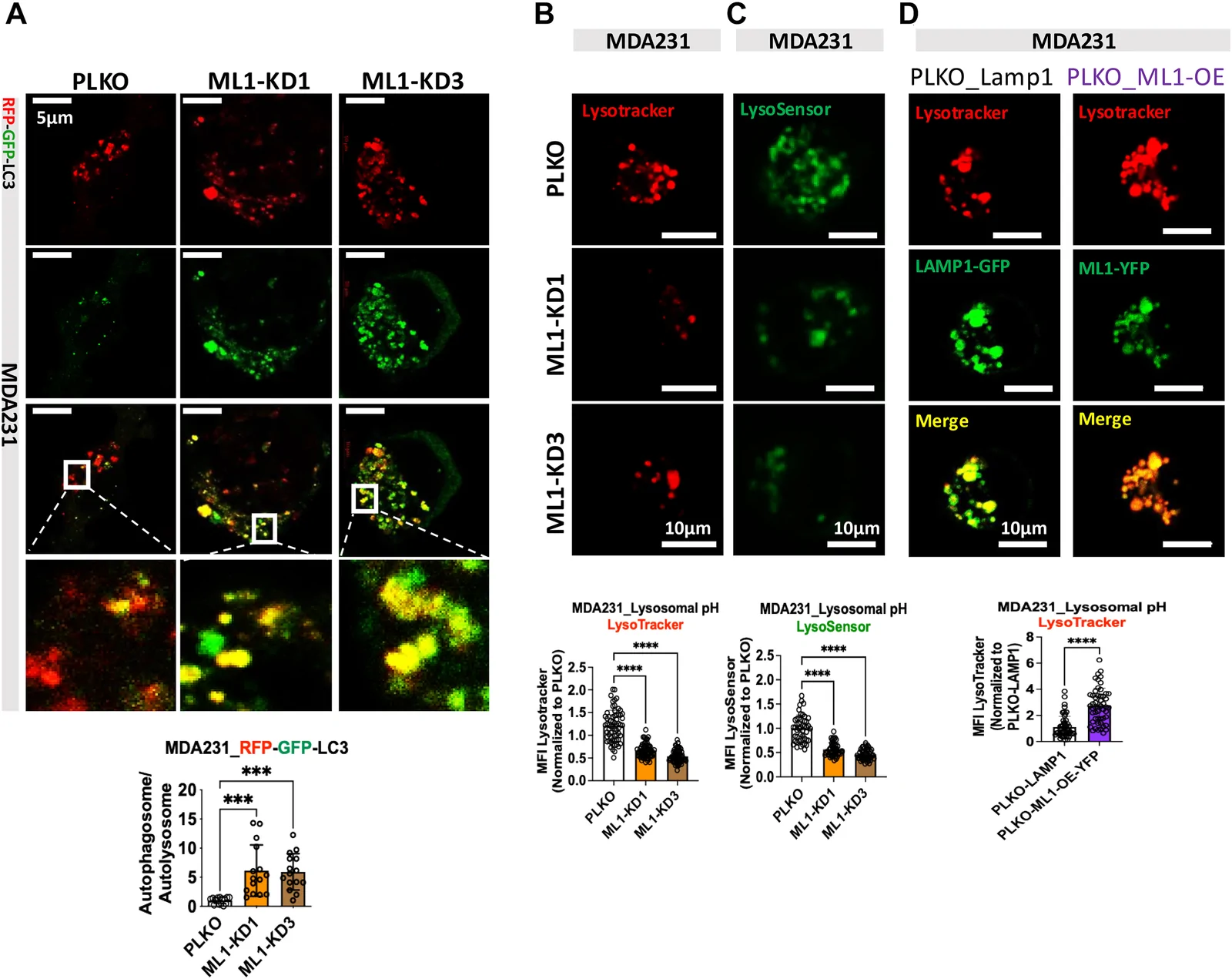
**TRPML1 knockdown impairs progression to acidic autolysosomal compartments and reduces lysosomal acidification in MDA-MB-231 TNBC cells.***A*, representative fluorescence images of MDA-MB-231 cells expressing tandem RFP-GFP-LC3 in PLKO and ML1-KD conditions. Individual RFP, GFP, and merged images are shown, with magnified regions highlighting LC3-positive puncta. *Yellow* puncta (RFP + GFP+) represent non-acidified LC3-positive compartments, whereas red-only puncta (RFP + GFP-) represent LC3-positive compartments exposed to an acidic environment. The accompanying graph shows the ratio of *yellow* to *red*-only puncta; Data are presented as mean ± SD from three independent biological experiments, with 15 cells analyzed per condition. Scale bar, 5 μm. *B*, representative confocal images of Lysotracker-stained PLKO and ML1-KD MDA-MB-231 cells, with corresponding quantification of LysoTracker fluorescence intensity per cell (with 64 cells analyzed per condition). *C*, representative confocal images of LysoSensor-stained PLKO and ML1-KD MDA-MB-231 cells, with corresponding quantification of LysoSensor fluorescence intensity per cell (with 47 images per condition). For *B* and *C*, data are presented as mean ± SD from 3 three independent biological experiments. Scale bar, 5 μm. *D*, representative confocal images of Lamp1-enrished and TRPML1-enriched PLKO MDA-MB-231 cells stained with Lysotracker. Cells positive for for GFP-Lamp1 or YFP-TRPML1 were used for LysoTracker analysis, and the corresponding graph shows Lysotracker fluorescence intensity per cell in Lamp1-GFP-PLKO and ML1-YFP-PLKO (PLKO-ML1-OE-YFP) cells. Data are presented as mean ± SD from three independent biological experiments, with 52 images per condition. Statistical significance was determined by one-way ANOVA with Tukey’s multiples comparison test for PLKO *versus* ML1-KD1/Ml1-KD3 MDA-MB-231 cells. ∗*p* < 0.05, ∗∗*p* < 0.01, and ∗∗∗*p* < 0.001. ns, not significant.

### TRPML1 knockdown impairs mitophagy and is associated with reduced TFEB nuclear localization in MDA-MB-231 cells

We next investigated whether the impaired autophagic flux and reduced mitochondria-lysosome proximity observed in ML1-KD MDA-MB-231 cells were accompanied by impaired mitochondrial clearance (48, 49). Using the mtKeima assay, a pH-sensitive ratiometric reporter that reports mitochondrial delivery to acidic lysosomal compartments (50), ML1-KD MDA-MB-231 cells displayed a significantly reduced red/green ratio compared to PLKO controls (Fig. 8*A*), consistent with reduced mitochondrial delivery to acidic lysosomal compartments and impaired mitophagy. Moreover, mitochondria (Mito-GFP) and LC3-positive autophagic vesicles (LC3-mCherry) showed reduced colocalization in ML1-KD cells (Fig. S3) (51, 52), further supporting reduced engagement of mitochondria with autophagic structures in MDA-MB-231 cells. We next examined the mitophagy-associated proteins PINK1, Parkin, and BNIP3, (53, 54). ML1-KD MDA-MB-231 cells exhibited markedly reduced PINK1 and Parkin levels, while BNIP3 levels remained unchanged (Fig. 8*B*), consistent with alteration of PINK1-Parkin-associated mitophagy machinery (55). PINK1 and Parkin levels were unchanged in MCF10A cells (Fig. 8*B*). Given the role of TFEB in lysosomal and mitochondrial quality control (56, 57), we next examined its subcellular localization. TFEB nuclear localization was significantly diminished in ML1-KD MDA-MB-231 cells compared with PLKO controls (Fig. 8*C*), whereas no change was observed in MCF10A cells (Fig. 8*D*). Furthermore, pharmacological activation of TRPML1 using ML-SA1 (10 μM) significantly enhanced TFEB nuclear translocation in MDA-MB-231 PLKO cells (Fig. 8*E*), supporting a positive relationship between TRPML1 activity and TFEB nuclear localization (58). Given previous reports linking CaM/calcineurin signaling to TRPML1-dependent TFEB regulation (58, 59), we also examined CaM abundance. CaM was modestly reduced in ML1-KD MDA-MB-231 cells (Fig. 8*F*). In parallel, proteins involved in mitochondrial biogenesis and stress adaptation, including PGC-1α (60), TFAM (61), and NRF2 (62), were also reduced in ML1-KD MDA-MB-231 cells (Fig. 8*F*). CaM, PGC-1α, TFAM, and NRF2 levels were unchanged in MCF10A cells (Fig. 8*F*). Together, these findings indicate that TRPML1 knockdown impairs mitophagy in MDA-MB-231 cells and is accompanied by reduced TFEB nuclear localization and alterations in mitochondrial maintenance and quality-control proteins.

**Figure 8:**
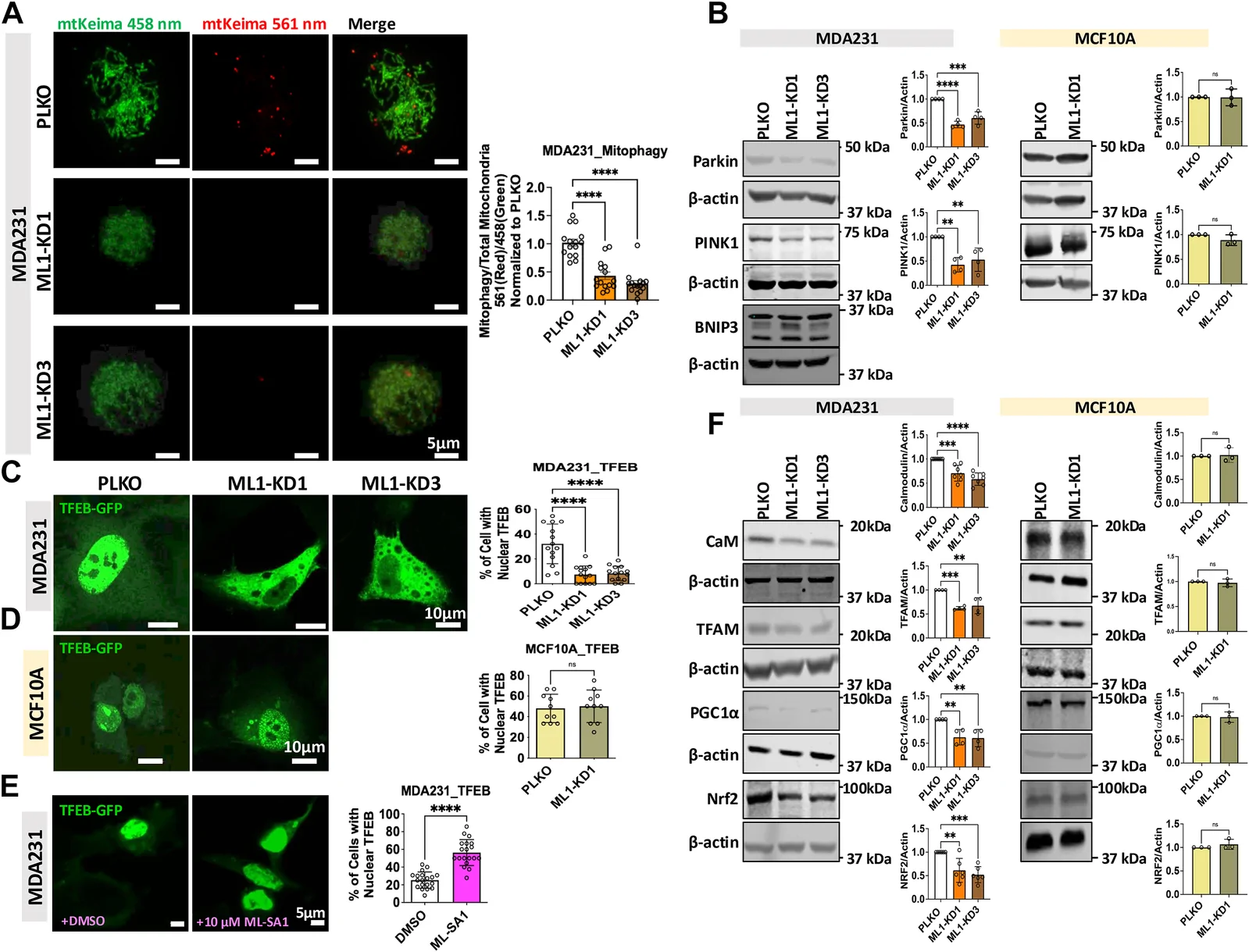
**TRPML1 knockdown impairs mitophagy and is associated with reduced TFEB nuclear localization in MDA-MB-231 cells.***A*, representative live-cell confocal images of mtKeima fluorescence in PLKO and ML1-KD mtKeima-stable MDA-MB-231 cells. Maximum-intensity projections are shown. The 458 nm excitation *green signal* represents mitochondria in a neutral environment, whereas the 561 nm excitation *red signal* represents mitochondria delivered to acidic lysosomal compartments. The corresponding graph shows the normalized 561/468-nm fluorescence. Data are presented as means ± SD from three independent biological experiments, with 15 cells analyzed per condition. Scale bar: 5 μm. *B*, representative Western blots of Parkin, PINK1, and BNIP3 in PLKO and ML1 KD of MDA-MB-231 and of Parkin and PINK1 in PLKO and ML1-KD1 MCF10A cells, with corresponding quantification (means ± SD) of Parkin (n = 4) and PINK1 (n = 4) in MDA-MB-231 cells and Parkin (n = 3) and PINK1 (n = 3) in MCF10A cells. *C*, representative confocal images of TFEB-GFP localization in PLKO and ML1-KD MDA-MB-231 cells, with corresponding quantifications of the % of cell displaying nuclear TFEB, with 14 images analyzed per condition. Scale bar: 5 μm (*D*) Representative confocal images and corresponding quantification TFEB-GFP localization in PLKO and ML1-KD1 MCF10A (10 images analyzed per condition), analyzed as described in *C*. Scale bar: 5 μm. *E*, representative confocal images of TFEB-GFP localization in MDA-MB-231 cells treated with DMSO or 10 μM ML-SA1, with corresponding quantification of the % of cells displaying nuclear TFEB (19 images analyzed per condition). Scale bar: 5 μm. *F*, representative Western blots and corresponding quantification of CaM (n = 8), TFAM (n = 4), PGC-1α (n = 4), and NRF2 (n = 6) in PLKO and ML1 KD of MDA-MB-231 cells, and CaM (n = 3), TFAM (n = 3), PGC-1α (n = 3), and NRF2 (n = 3) in PLKO and ML1-KD1 MCF10A cells. Western blot quantifications were normalized to β-Actin and expressed relative to the corresponding PLKO. Data are presented as mean ± SD, with n representing independent experiments. Statistical significance was determined by one-way ANOVA with Tukey’s multiple comparison test for PLKO *versus* ML1-KD1/Ml1-KD3 of MDA-MB-231, and unpaired *t* test and Mann–Whitney *U* test for PLKO and ML1-KD1 of MCF10A cells. ∗*p* < 0.05, ∗∗*p* < 0.01, and ∗∗∗*p* < 0.001. ns, not significant.

### TRPML1 knockdown alters cytosolic and mitochondrial Ca^2+^ responses

Having established that TRPML1 loss compromises mitochondrial quality control, with impaired autophagic clearance, reduced mitophagy, and reduced TFEB nuclear localization, we next asked whether TRPML1 channel activity also influences mitochondrial Ca^2+^ responses. This was particularly relevant because TRPML1-mediated lysosomal Ca^2+^ release has been proposed to support Ca^2+^ transfer to mitochondria at organelle contact sites (21), and ML1-KD cells displayed altered mitochondria–lysosome and mitochondria–ER proximity (Fig. 5). We therefore tested whether pharmacological activation of TRPML1 was sufficient to elicit a mitochondrial Ca^2+^ response (Fig. 9*A*). To this end, we activated TRPML1 with ML-SA1 and monitored mitochondrial Ca^2+^ responses using Rhod-2, first in the absence of extracellular Ca^2+^. Even at 50 μM (a high concentration), ML1-SA1 failed to induce a detectable mitochondrial Ca^2+^ response in either MDA-MB-231 or MCF10A cells when extracellular Ca^2+^ was absent, regardless of TRPML1 expression status (PLKO or ML1-KD) (Fig. 9*B*). Similarly, overexpression of TRPML1 in MDA-MB-231 cells did not produce a detectable increase in mitochondrial Ca^2+^ response under Ca^2+^-free conditions (Fig. 9*C*). To determine whether TRPML1 activation could nevertheless mobilize intracellular cytosolic Ca^2+^, we monitored cytosolic Ca^2+^ using Fura-2AM under the same Ca^2+^-free conditions. ML1-SA1 at 50 μM did not affect cytosolic Ca^2+^ in PLKO and ML1-KD MDA-MB-231 cells expressing endogenous levels of TRPML1 (Fig. 9*D*). However, when TRPML1 was overexpressed in PLKO MDA-MB-231 cells, a significant increase in cytosolic Ca^2+^ was detected (Fig. 9, *E* and *F*), indicating that TRPML1-mediated intracellular Ca^2+^ release was detectable under conditions of increased channel abundance. Importantly, this cytosolic Ca^2+^ response was not accompanied by a detectable mitochondrial Ca^2+^ response (Fig. 9*C*), providing no evidence for readily detectable direct lysosome-to-mitochondria Ca^2+^ transfer under these experimental conditions. We next repeated these measurements in the presence of extracellular Ca^2+^. Under these conditions, exposure to ML1-SA1 led to a dose-dependent increase in mitochondrial Ca^2+^ responses in PLKO MDA-MB-231 cells, most evident at 25 to 50 μM (Fig. 9, *G1–G5*). ML1-KD MDA-MB-231 cells displayed significantly enhanced mitochondrial Ca^2+^ responses that were detectable at the lower ML1-SA1 concentration of 5 μM (Fig. 9, *G2–G5*), indicating enhanced Ca^2+^ responsiveness following TRPML1 knockdown. Under the same extracellular Ca^2+^-containing conditions, ML1-SA1 caused a greater increase in cytosolic Ca^2+^ in ML1-KD than in PLKO cells (Fig. 9, *H* and *I*). Thus, the enhanced mitochondrial Ca^2+^ response in ML1-KD cells occurred in parallel with an enhanced cytosolic Ca^2+^ response when extracellular Ca^2+^ was available. Finally, to determine whether the enhanced mitochondrial Ca^2+^ responsiveness of ML1-KD cells extended beyond ML-SA1 exposure, we examined the response to physiological receptor stimulation with ATP. ATP produced significantly greater mitochondrial Ca^2+^ responses in ML1-KD than in PLKO MDA-MB-231 cells (Fig. 9, *J* and *K*). The enhanced response occurred without changes in mitochondrial Ca^2+^ uniporter (MCU) abundance (Fig. 9, *L* and *M*) or mitochondrial membrane potential (Fig. 9*N*). Together, these findings show that TRPML1 knockdown enhances cytosolic and mitochondrial Ca^2+^ responses under extracellular Ca^2+^-containing conditions, including mitochondrial Ca^2+^ responsiveness to ATP stimulation, without evidence for direct lysosome-to-mitochondria Ca^2+^ transfer under the conditions tested.

**Figure 9:**
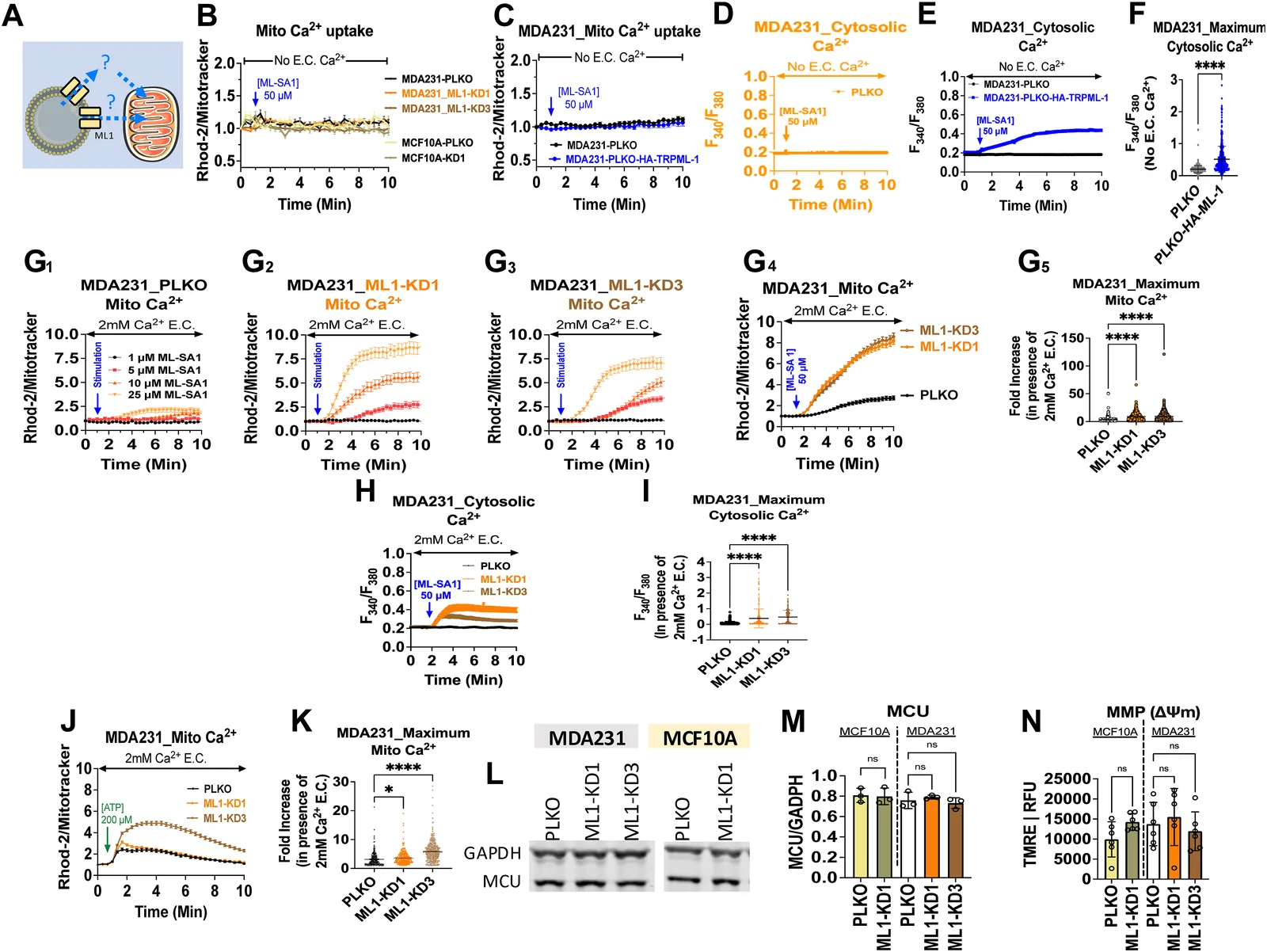
**TRPML1 knockdown enhances extracellular Ca^2+^-dependent cytosolic and mitochondrial Ca^2+^ responses in MDA-MB-231 cells.***A*, schematic illustrating the experimental question of whether TRPML1-dependent lysosomal Ca^2+^ release directly contributes to mitochondrial Ca^2+^ responses or acts through an intermediary cytosolic pathway. *B*, mitochondrial Ca^2+^ response kinetics following stimulation with 50 μM ML-SA1 in the absence of extracellular Ca^2+^ in the indicated PLKO and ML1-KD MDA-MB-231 and MCF10A conditions. *C*, mitochondrial Ca^2+^ response kinetics following stimulation with 50 μM ML-SA1 in MDA-MB-231 PLKOs and HA-TRPML1-overexpressing (ML1-OE) cells in the absence of extracellular Ca^2+^. *D*, cytosolic Ca^2+^ response kinetics following stimulation with 50 μM ML-SA1 in PLKO and ML1-KD MDA-MB-231 cells in the absence of extracellular Ca^2+^. *E*, cytosolic Ca^2+^ response kinetics following stimulation with 50 μM ML-SA1 in MDA-MB-231 PLKO and ML1-OE cells in the absence of extracellular Ca^2+^. *F*, corresponding quantification of the peak cytosolic Ca^2+^ response shown in (*E*). (*G1–G3*) Mitochondrial Ca^2+^ response kinetics to increasing concentrations of ML1-SA1 (1-25 μM) in PLKOs (*G1*), ML1-KD1 (*G2*), and ML1-KD3 (*G3*) MDA-MB-231 cells in the presence of 2 mM extracellular Ca^2+^. (*G4*) Mitochondrial Ca^2+^ response kinetics following stimulation with 50 μM ML1-SA1 in PLKOs and ML1-KD MDA-MB-231 cells in the presence of 2 mM extracellular Ca^2+^. (*G5*) Corresponding quantification of the peak mitochondrial Ca^2+^ response shown in (*G4*). *H*, cytosolic Ca^2+^ response kinetics following stimulation with 50 μM ML1-SA1 in PLKO and ML1-KD MDA-MB-231 cells in the presence of 2 mM extracellular Ca^2+^. *I*, corresponding quantification of the peak cytosolic Ca^2+^ response shown in (*H*). *J*, mitochondrial Ca^2+^ response kinetics following stimulation with 200 μM ATP in PLKO and ML1-KD MDA-MB-231 cells in the presence of 2 mM extracellular Ca^2+^. *K*, corresponding quantification of the peak mitochondrial Ca^2+^ response shown in (*J*). *L*, representative Western blots of MCU in PLKO and ML1-KD MDA-MB-231 and MCF-10A cells. *M*, corresponding quantification of MCU normalized to GAPDH (n = 3 for both MDA-MB-231 and MCF10A cells). *N*, quantification of mitochondrial membrane potential using TMRE measurements in PLKO and ML1-KD MDA-MB-231 and MCF-10A cells. Mitochondrial Ca^2+^ responses were measured using Rhod-2 AM and normalized to MitoTracker *Green*. Fluorescence; Cytosolic Ca^2+^ responses were measured with Fura-2 AM. All experiments represent three independent biological replicates (n = 3). Statistical significance was determined by a Mann–Whitney *U* test for *panel F*; Kruskal–Wallis test with Dunn’s multiple-comparisons test for *panels G5*, *I*, and *K*; and one-way ANOVA with Tukey’s multiple-comparisons test for *panels M* and *N*. ∗*p* < 0.05, ∗∗*p* < 0.01, ∗∗∗*p* < 0.001 and ∗∗∗∗*p* < 0.0001, ns, not significant.

### TRPML1 knockdown induces broad metabolism remodeling in MDA-MB-231 cells

We next asked whether the mitochondrial dysfunction observed in ML1-KD was accompanied by metabolic remodeling. To address this, we performed targeted mass spectrometry-based metabolomics in MDA-MB-231 and MCF10A cells. ML1-KD induced only minimal changes in the overall metabolomic profile of MCF10A cells compared with PLKO controls (Fig. 10*A*). By contrast, ML1-KD MDA-MB-231 cells showed broader alterations involving the tricarboxylic acid (TCA) cycle, amino acid metabolism, nucleotide metabolism, and carnitine species (Fig. 10). Within the TCA cycle, ML1-KD MDA-MB-231 cells showed a marked reduction in oxoglutaric acid (OGA), accompanied by increased 2-hydroxyglutarate (2-HG), isocitrate and succinate (Fig. 10*B*). This pattern is consistent with remodeling of TCA-associated metabolite pools around the OGA node rather than a uniform reduction in TCA-cycle-associated metabolites. In parallel, amino acid metabolism was remodeled in ML1-KD MDA-MB-231 cells with L-glutamic acid and L-proline increased and L-glutamine and L-arginine were reduced (Fig. 10*C*). These changes are consistent with altered glutamine/glutamate-associated amino acid metabolism. Nucleotide metabolism was similarly affected with increased GTP, inosine and xanthosine, whereas the dGTP was reduced (Fig. 10*D*). These changes indicate altered nucleotide pools following ML1-KD. Finally, carnitine species were among the most strongly increased metabolites in ML1-KD MDA-MB-231 cells (Fig. 10*E*). Given the role of carnitine in mitochondrial fatty acid transport and β-oxidation (63, 64), the accumulation of these species is consistent with altered fatty acid handling and is in line the reduced FAO capacity observed by CENCAT (Fig. 3*G*).

**Figure 10:**
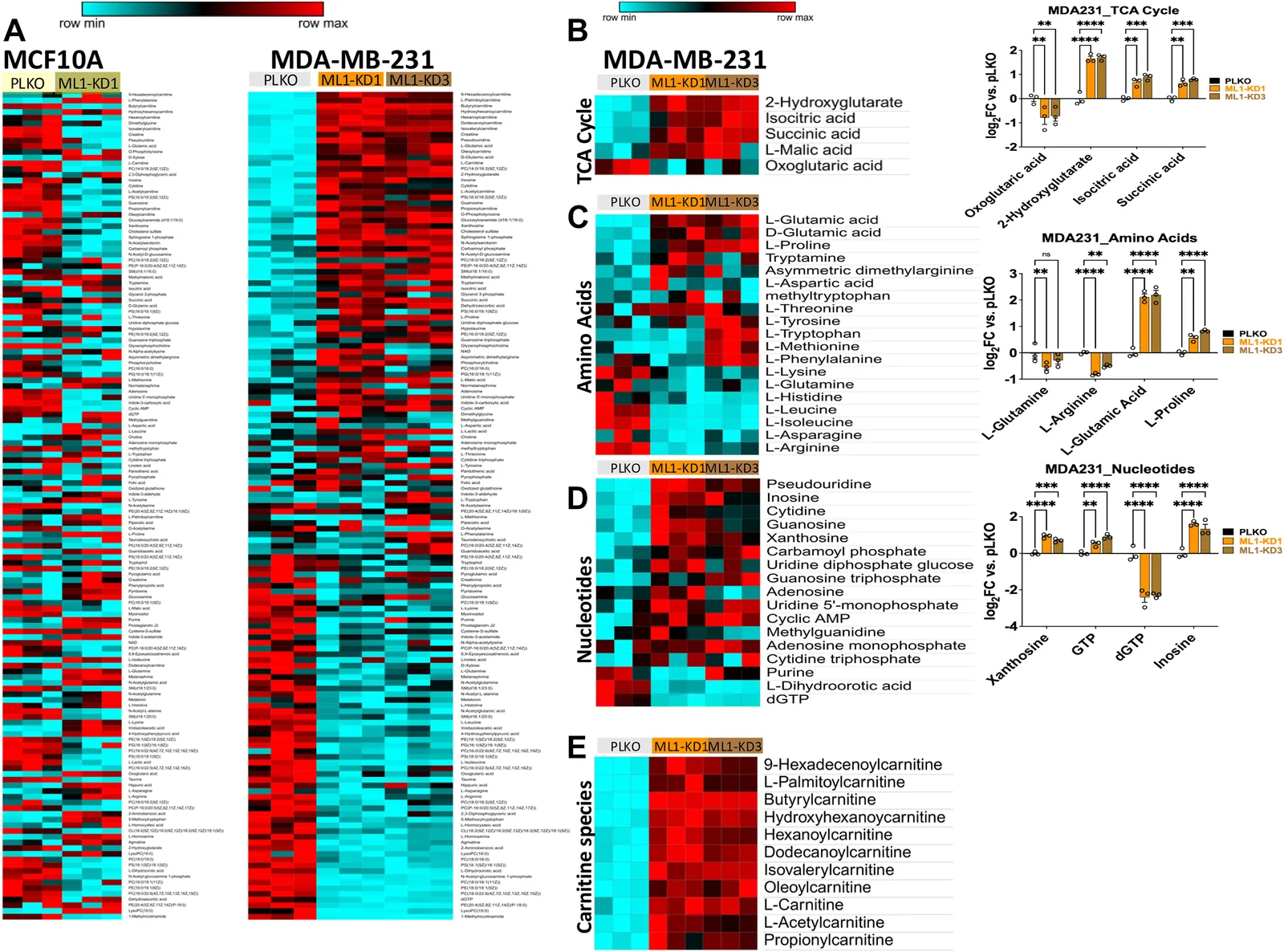
**TRPML1 knockdown remodels TCA-cycle, amino acid, nucleotide, and carnitine metabolite pools in MDA-MB-231 cells.** Targeted mass spectrometry-based metabolomic profiling was performed in MDA-MB-231 and MCF10A cells under PLKO and ML1-KD conditions. *A*, heatmap showing the relative changes in the measured metabolite profiles. *B–D*, heatmaps and corresponding bar graphs showing selected metabolites associated with the TCA cycle (*B*), amino acid metabolism (*C*), and nucleotide metabolism (*D*) in PLKO and ML1-KD MDA-MB-231 cells. *E*, heatmap of carnitine species showing relative abundance changes in PLKO and ML1-KD MDA-MB-231 cells. Data were acquired from one biological experiment analyzed in technical triplicate. Statistical significance was determined by one-way ANOVA with Tukey’s multiple comparison test for PLKO *versus* ML1-KD1/Ml1-KD3 of MDA-MB-231. ∗*p* < 0.05, ∗∗*p* < 0.01, and ∗∗∗*p* < 0.001. ns, not significant.

### Genetic and pharmacological inhibition of TRPML1 enhances the responses to doxorubicin and paclitaxel in MDA-MB-231 cells

To determine whether the metabolic disruption, bioenergetic impairment, and mitochondrial dysfunction observed in ML1-KD MDA-MB-231 cells were associated with altered chemotherapy responsiveness, we investigated whether TRPML1 depletion increases the sensitivity to doxorubicin (DOXO) and paclitaxel (PXL) (Fig. 11*A*). First, we performed a DOXO and PXL dose-response analyses in MDA-MB-231 PLKO control cells and identified 3 nM as a subeffective concentration that did not significantly alter cell number (Fig. 11*B*). Next, we assessed whether ML1-KD MDA-MB-231 cells were more responsive to 3 nM DOXO and PXL. At 3 nM, both DOXO and PXL significantly reduced cell number in ML1-KD MDA-MB-231 cells, whereas neither treatment significantly affected PLKO control cells (Fig. 11*C*). We next asked whether pharmacological inhibition of TRPML1 could similarly enhance the response to these subeffective chemotherapy concentrations Consistent with our previous findings, ML-SI1 alone reduced MDA-MB-231 cell number, and co-treatment with 3 nM DOXO or PXL further reduced cell number compared to ML-SI1 alone (Fig. 11*D*). Together, these findings show that both genetic and pharmacological inhibition of TRPML1 enhance MDA-MB-231 cell responses to otherwise subeffective concentrations of DOXO and PXL, providing orthogonal pharmacological support for increased chemotherapy responsiveness following TRPML1 inhibition *in vitro*.

**Figure 11:**
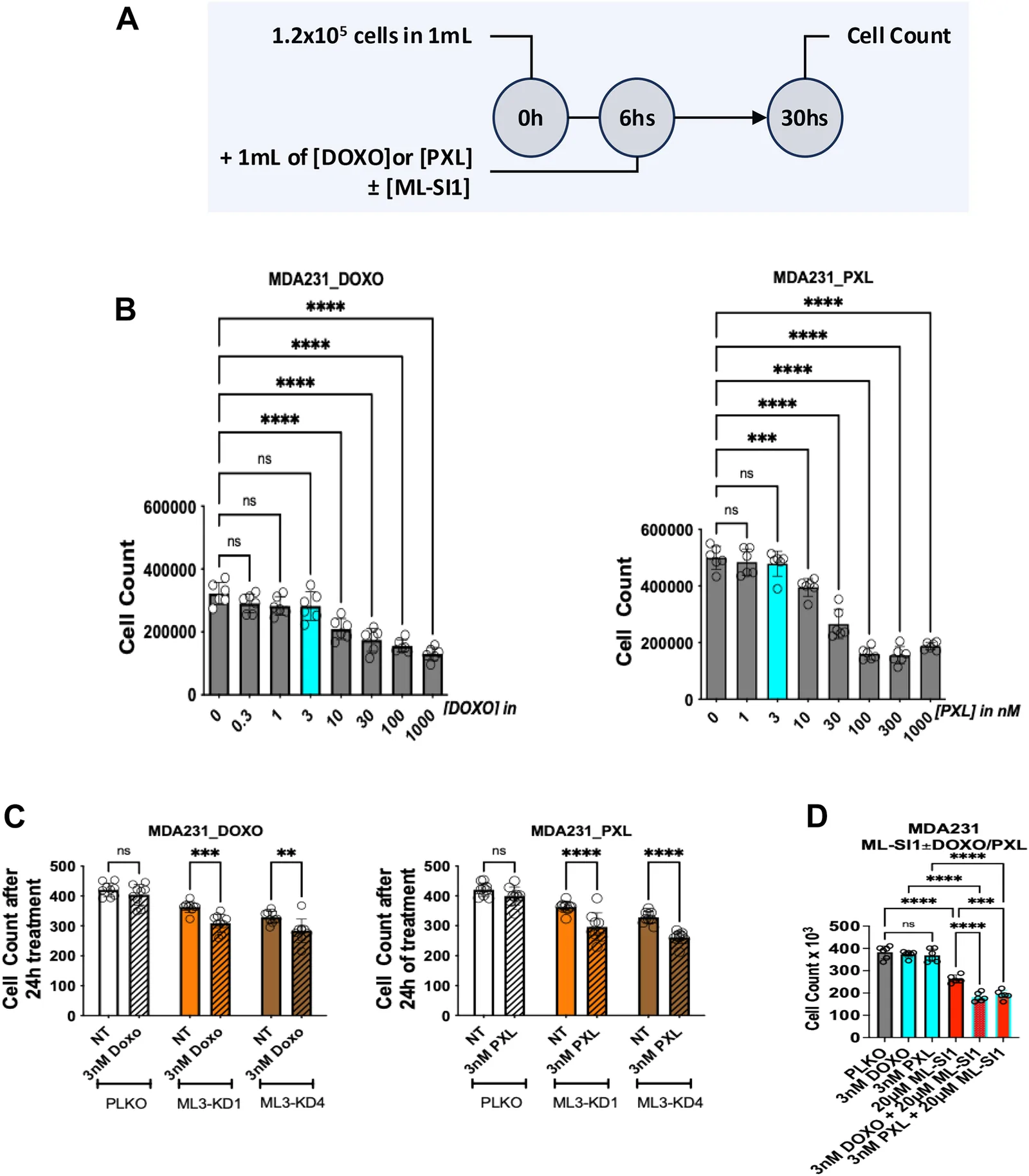
**Genetic and pharmacological inhibition of TRPML1 enhances responses to doxorubicin and paclitaxel in MDA-MB231 cells.***A*, schematic of the experimental protocol. MDA-MB-231 cells were seeded at 1.2 × 105 cells in 1 ml medium; 6 h later, 1 ml medium containing the indicated treatments was added, and cells were counted at 30 h. *B*, Dose–response of DOXO and PXL, identifying 3 nM as the lowest dose having no effect on MDA-MB-231 PLKO cells, for both doxorubicin (DOXO) and paclitaxel (PXL) in MDA-MB-231 cells, identifying 3 nM as a subeffective concentration of each agent that did not significantly alter cell number. *C*, cell number in PLKO ML1-KD1 and ML1-KD3 MDA-MB-231 cells treated with vehicle, 3 nM DOXO or 3 nM PXL. *D*, cell number in MDA-MB-231 PLKO cells following pharmacological inhibition of TRPML1 with ML-SI1 alone, or in combination with 3 nM DOXO or PXL. ML-SI1 alone reduced cell number, whereas addition of DOXO or PXL further reduced cell number compared with ML-SI1 alone. Data were acquired from 3 independent biological experiments, with 6 technical replicates per condition in each experiment and are presented as mean ± SD. Statistical significance was determined by one-way ANOVA with Tukey’s multiples comparison test. ∗*p* < 0.05, ∗∗*p* < 0.01, and ∗∗∗*p* < 0.001. ns, not significant.

## Discussion

Here, we identify TRPML1 as an important regulator of lysosome–mitochondria communication that supports mitochondrial homeostasis, metabolic fitness, and cell growth in MDA-MB-231 TNBC cells. Our findings support a model in which TRPML1 loss is associated with reduced lysosomal acidification, uncoupling of autophagy initiation from degradative flux, and impaired mitochondrial quality control accompanied by reduced TFEB nuclear localization. These changes occur together with mitochondrial fragmentation, remodeling of mitochondria–lysosome and mitochondria–ER proximity, enhanced cytosolic and mitochondrial Ca^2+^ responses, oxidative stress, and impaired bioenergetic capacity. This compromised mitochondrial state is accompanied by broad metabolic remodeling, G0/G1 arrest, and caspase-3/7-independent cell death (Fig. 12). Importantly, many of these alterations were not observed in non-cancerous MCF10A cells, and both genetic and pharmacological inhibition of TRPML1 enhanced the response of MDA-MB-231 cells to otherwise subeffective concentrations of doxorubicin and paclitaxel.

**Figure 12:**
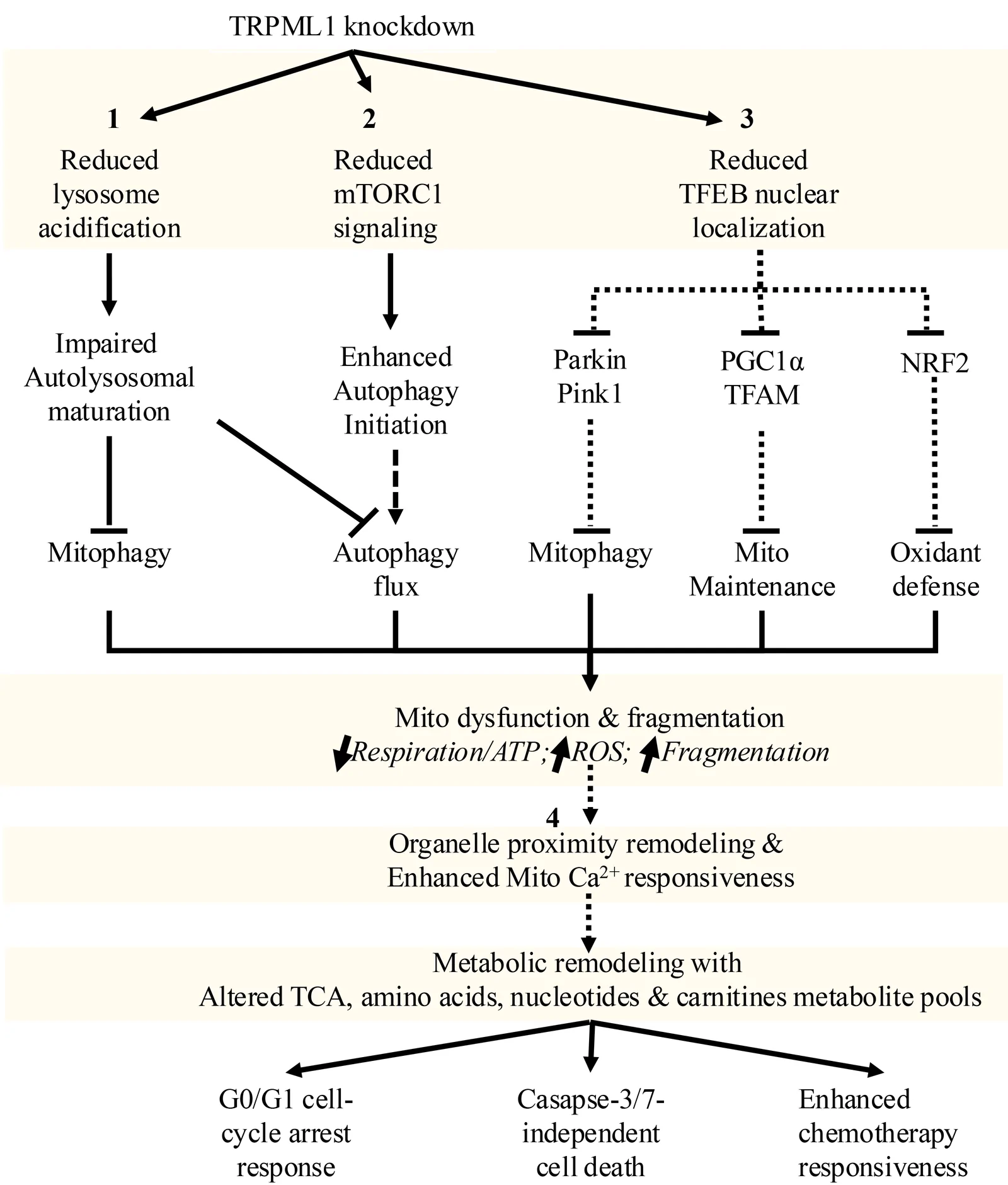
**Proposed model of TRPML1-dependent lysosome-mitochondria homeostasis in MDA-MB-231. TRPML1 knockdown is associated with** (1) **reduced lysosomal acidification** (2)**, reduced mTORC1 signaling, and** (3) **reduced TFEB nuclear localization.** These alterations occur together with mitochondrial dysfunction and fragmentation, characterized by reduced respiration and ATP production and increased ROS (4). This mitochondrial phenotype is accompanied by organelle-proximity remodeling and enhanced mitochondrial Ca^2+^ responsiveness. These changes are further associated with metabolic remodeling involving TCA-cycle-associated metabolites, amino acids, nucleotide pools, and carnitine species, together with G0/G1 cell-cycle arrest, caspase-3/7-independent cell death, and enhanced responsiveness to doxorubicin and paclitaxel.

A central mechanistic insight from our study is that TRPML1 loss dissociates signaling associated with autophagy initiation from downstream autophagic flux in MDA-MB-231 cells. ML1-KD reduces mTORC1 signaling while enhancing AMPK–ULK1 signaling, a pattern consistent with increased autophagy-initiation signaling. TRPML1-dependent lysosomal Ca^2+^ release has been linked to CaM-dependent mTORC1 activation (65), suggesting that loss of this input could contribute to reduced mTORC1-mediated inhibition of ULK1; in parallel, the mitochondrial ATP deficit induced by ML1-KD may promote AMPK activation and further reinforce ULK1 (66, 67). However, TRPML1 loss also reduces lysosomal acidification, likely through impaired V-ATPase-dependent proton handling (68, 69). Since lysosomal acidity is essential for autophagosome–lysosome fusion, hydrolase activity, and cargo degradation, reduced acidification is consistent with the impaired autophagic flux observed in ML1-KD MDA-MB-231 cells (44, 45, 46). Thus, TRPML1-deficient MDA-MB-231 cells enter an “initiated-but-incomplete” autophagy state, in which signaling associated with autophagy initiation is enhanced while lysosomal dysfunction limits completion of the degradative process.

Beyond impaired autophagic flux, TRPML1 loss also disrupts mitochondrial quality-control pathways. In ML1-KD cells, reduced CaM abundance was accompanied by reduced TFEB nuclear localization, in line with the established model in which TRPML1-mediated lysosomal Ca^2+^ release activates calcineurin-dependent TFEB dephosphorylation and nuclear entry (58). Reduced TFEB nuclear localization occurred in the parallel with decreased PINK1 and Parkin levels, two central components of canonical mitophagy, while the unchanged BNIP3 levels suggest that mitophagy pathways are not uniformly affected (70, 71). TFEB also supports mitochondrial biogenesis and stress adaptation through PGC-1α- (72), TFAM- (73), and NRF2- (73) associated pathways. Their reduction in ML1-KD cells is therefore consistent with broader impairment of mitochondrial maintenance and stress adaptation. In parallel, ML1-KD reduced MFN1, MFN2, and OPA1 without detectable changes in the examined fission markers (74). Because MFN1/MFN2 and OPA1 mediate outer- (75) and inner-membrane fusion (76), respectively, their reduction may contribute to fragmented mitochondrial morphology observed in ML1-KD MDA-MB-231 cells.

Another major finding of our study is that TRPML1 loss alters mitochondrial positioning within the organellar landscape. In ML1-KD MDA-MB-231 cells, the smaller, fragmented mitochondria phenotype is accompanied by increased mitochondria–ER proximity and reduced mitochondria–lysosome proximity. We propose that mitochondrial fragmentation and reduced volume may favor redistribution of mitochondrial within the ER network, contributing to this altered special organization. Because ER–mitochondria contacts are important sites of Ca^2+^ exchanges, this spatial reorganization may provide a structural context for the altered mitochondrial Ca^2+^ handling observed in ML1-KD cells (21, 77, 78), although the functional contribution of these contacts remains to be established. ML1-KD cells displayed enhanced mitochondrial Ca^2+^ responses when extracellular Ca^2+^ was available or following ATP stimulation, indicating that the enhanced mitochondrial response occurs in the context of increased cytosolic Ca^2+^ availability rather than detectable direct lysosomal Ca^2+^ transfer. Indeed, although a previous study proposed that TRPML1 can deliver lysosomal Ca^2+^ directly to adjacent mitochondria through contact sites (21), our data do not support this mechanism under the conditions tested. TRPML1 activation by ML-SA1 under Ca^2+^-free conditions failed to increase mitochondrial Ca^2+^ even when TRPML1 was overexpressed, although the same conditions increased cytosolic Ca^2+^, indicating that intracellular TRPML1-dependent Ca^2+^ mobilization can occur without a corresponding mitochondrial Ca^2+^ response.

These upstream alterations are accompanied by a metabolically constrained state in ML1-KD cells. Rather than a uniform metabolic collapse, the metabolic signature points to remodeling of interconnected pathways supporting mitochondrial metabolism, biosynthesis, and proliferation. OGA depletion, together with increased 2-HG, isocitrate, and succinate, is consistent with remodeling of TCA-associated metabolite pools centered on the OGA node rather than uniform suppression of the TCA cycle (79, 80); In parallel, reduced glutamine and arginine together with increased glutamate and proline are consistent with remodeling of glutamine/glutamate-associated metabolism, while reduced dGTP alongside increased purine-related metabolites is consistent with altered nucleotide homeostasis and the reduced proliferative state of ML1-KD cells (81, 82). Similarly, selective dGTP depletion alongside accumulation of other purine metabolites is consistent with altered nucleotide homeostasis and the reduced proliferative state of ML1-KD cells (83, 84). Finally, carnitine species accumulated markedly in ML1-KD MDA-MB-231 cells. Carnitine supports mitochondrial fatty-acid import and β-oxidation (85, 86), and elevated carnitine has been associated with poorer breast cancer survival (87, 88). In our model, however, FAO utilization was reduced, indicating that carnitine accumulation reflects altered fatty-acid handling rather than enhanced lipid oxidation.

A major functional finding of our study is that TRPML1 loss increases chemotherapy responsiveness in MDA-MB-231 cells. This phenotype is consistent with the broader disruption of stress-adaptation pathways observed in ML1-KD cells, particularly because mTOR/autophagy signaling has been implicated in doxorubicin resistance, while mitochondrial dynamics and dysfunction influence therapeutic response in TNBC and other cancer models (89, 90, 91, 92, 93). The increased responsiveness to both doxorubicin and paclitaxel therefore suggests that TRPML1 supports a cellular capacity to withstand chemotherapy rather than a drug-specific resistance mechanism. Importantly, the further reduction in cell number produced by combining ML-SI1 with otherwise subeffective concentrations of DOXO or PXL provides complementary pharmacological support for this TRPML1-dependent vulnerability. In this context, we recently reported that depletion of the related lysosomal channel TRPML3 also enhanced responses to subeffective DOXO and PXL concentrations in MDA-MB-231 and HCC1806 cells (31). Despite this shared phenotype, TRPML1 and TRPML3 operate within distinct lysosomal pH environments: TRPML1 is active at acidic lysosomal pH (94, 95), whereas TRPML3 is proton-inhibited and becomes permissive as lumen pH rises (96, 97). Consistent with this divergence, ML1-KD reduces lysosomal acidification, suppresses mTORC1 signaling, and uncouples autophagy-initiation signaling from degradative flux, whereas ML3-KD increases lysosomal acidification, activates mTORC1, and suppresses autophagy (31). These distinct lysosomal responses converge on increased chemotherapy responsiveness, supporting nonredundant roles for TRPML1 and TRPML3 in TNBC stress adaptation.

Our study has some limitations. Although the growth phenotype was reproduced in HCC1806 and HS578T TNBC cells, and our prior work demonstrated TRPML1-dependent TNBC growth *in vivo* (27), the lysosome-mitochondria signaling architecture characterized here was established primarily in MDA-MB-231 cells and remains to be validated in additional TNBC models. Several mechanistic links remain unresolved, including the relationship between CaM/TFEB signaling and changes in PINK1/Parkin, whether reduced MFN1/2 and OPA1 levels translate into impaired mitochondrial fusion, and the mechanism underlying the enhanced mitochondrial Ca^2+^ responsiveness, including the functional contribution of increased ER-mitochondria proximity. Finally, although both genetic and pharmacological TRPML1 inhibition enhanced responses to DOXO and PXL *in vitro*, future rescue and *in vivo* combination studies could further establish target specificity and therapeutic relevance.

In conclusion, our study extends the role of TRPML1 in TNBC beyond its previously defined functions in mTORC1 signaling, lysosomal ATP release, tumor growth, and invasion by identifying TRPML1 as an important regulator of lysosome–mitochondria homeostasis in MDA-MB-231 cells. TRPML1 loss uncouples autophagy initiation from degradative flux, impairs mitochondrial quality control and bioenergetics, and is accompanied by broad metabolic remodeling. Importantly, both genetic and pharmacological inhibition of TRPML1 enhance responses to otherwise subeffective concentrations of DOXO and PXL *in vitro*. Together, these findings support TRPML1-dependent lysosomal signaling as an important contributor to mitochondrial-metabolic resilience and chemotherapy response in this TNBC model.

## Experimental procedures

### Cell culture

TNBC cell lines (MDA-MB-231, HCC1806, HS578T) and non-cancerous control MCF10A cell line were kindly provided by Dr Marcato Paola (Dalhousie University), while HEK293T cell line were generously provided by Dr Paul Linsdell (Dalhousie University). All cells were routinely tested for *mycoplasma* contamination. MDA-MB-231 and HEK293T cells were cultured in DMEM supplemented with 10% FBS. HCC1806 cells in RPMI-1640 supplemented with 10% FBS. HS578T cells in DMEM supplemented with 10% fetal bovine serum (FBS), and 10 μg/ml insulin. MCF10A cells in DMEM/F-12 supplemented 5% horse serum, 10 μg/ml insulin, 20 ng/ml human epidermal growth factor (hEGF), and 0.5 μg/ml hydrocortisone.

### Lentivirus production, stable transduction, and transient infection

Short hairpin RNA (shRNA) clones targeting TRPML1 were purchased from Dharmacon. An empty PLKO plasmid and two different shTRPML1 constructs (ML1-KD1 and ML1-KD3) were used according to the protocol for the third generation lentiviral packaging system (98). Briefly, lentiviral particles were generated in HEK293T cells by co-transfection of psPAX2 (6 μg) and pMD2G (3 μg), with either the PLKO or an ML1-KD plasmid (6 μg) using Lipofectamine 2000 (Thermo scientific). Lentivirus supernatants were collected at 48 h post-transfection, filtered (Millex-GS; 0.45-μm sterile filter), and stored at −80 °C. For transduction, MDA-MB-231, HCC1806, HS578T, and MCF10A cells were seeded in 6-well plates and cultured for 24 h. Medium containing 200 μl of lentivirus and 8 μg/ml of polybrene (Sigma) was added to the cells and allowed to incubate for 48 h, then selected by Puromycin (2 μg/ml) for 4 days. Knockdown efficiency was assessed by RT-qPCR. For this, total RNA was extracted using TRIzol reagent (Invitrogen) and purified with the DNA-free DNA Removal Kit (Thermo Fisher Scientific). Complementary DNA (cDNA) was synthesized from 2 μg RNA using iScript Reverse Transcriptase (Bio-Rad). Quantitative PCR (qPCR) was performed using SYBR Green Mater Mix (Bio-Ras) and gene-specific primers. For transient transfection, cells were transfected with RFP-GFP-LC3, TFEB-GFP, Mito-GFP, or Lamp1-GFP plasmids using Lipofectamine 2000 (Thermo Fisher Scientific) and imaged or analyzed 24 to 48 h post-transfection.

### Cell proliferation, colony formation, and chemotherapy-response assays

For cell proliferation assay, cells were seeded at a density of 5 × 10^5^ cells/well and incubated for 72 h at 37 °C/5% CO_2_. After incubation, cells were trypsinized and counted using automated cell counter (CellDrop BF, DeNovix). For the colony formation assay, 500 cells were seeded per well in 6-well plates, and cultured for 14 days with medium changed every 72 h. On day 14, the medium was removed, and cells were gently washed with PBS. Colonies were fixed and stained with 1 ml of Coomassie Brilliant Blue (R-250, #1610400, Bio-Rad) dissolved in methanol and Acetic Acid, for 20 min at RT. Plates were rinsed under running water for 3 min and allowed to air-dry. Colony images were captured using a digital camera, and the colony formation was quantified by counting the number of colonies using Image J software. For drug tolerance assay, PLKO control and TRPML1 KDs cells, were seeded in 35-mm culture dishes at a density of 1.2 × 10^5^ cells per dish in complete media. Cells were allowed to adhere for 6 h at 37 °C in a humidified incubator with 5% CO_2_. After attachment, cells were treated with either Doxorubicin or Paclitaxel in presence or absence of ML-SI1. Following 24 h of drug exposure, cells were harvested using trypsinization, and total cell numbers were determined using an automated cell counter.

### Western blot analysis

PLKO and ML1-KD of MDA-MB-231 and MCF10A cells were lysed in radioimmunoprecipitation assay (RIPA) buffer supplemented with protease and phosphatase inhibitors (Thermofisher Scientific). Protein concentrations were determined using the bicinchoninic acid (BCA) assay (Thermofisher Scientific). Equal amounts of protein were mixed with 4 × Laemmli buffer, boiled, and resolved on SDS-PAGE gels. Proteins were transferred to nitorcellulose membranes and blocked for 1 h in PBS containing 0.1% Tween-20 and 5%BSA. Membranes were incubated overnight at 4C with the following primary antibodies (all from Cell Signaling Technology, 1:1000 dilution unless otherwise noted): Cell cycle regulators: anti-Cyclin B1 (#4138, anti-Cyclin D1(#2922), anti-p27 (#3686), anti-E2F1 (#3742); Apoptosis markers: anti-Cleaved-Caspase 9(#7237), anti-Caspase 9 (#9502), anti-Cleaved-Caspase7 (#9491), anti-Caspase7 (#9492), anti-Cleaved-Caspase3 (9661), anti-Caspase3 (#9662), anti-p-Bcl-2(S70) (#2827), anti-EndoG(#4969), anti-AIF(#4642), Mitochondrial Dynamics: anti-MFN1 (#14739), anti-MFN2 (#11925), anti-OPA1 (#80471), anti-p-DRP1 (S616) (#3455), anti-p-DRP1 (S637) (#4867), anti-MFF (#86668), Mitochondrial bioenergetics: OXPHOS (#ab110411, Abcam), MCU (#14997), Autophagy regulator: anti-p-P70S6K (Thr389) (#9205, Cell Signaling), anti-P70S6K (#9202, Cell Signaling), anti-p-ULK1 (S757) (#6888, Cell Signaling), anti-p-ULK1 (S555) (#5869, Cell Signaling), anti-ULK1 (#8054, Cell Signaling), anti-LC3 A/B (#12741, Cell Signaling), anti-p-AMPK (Thr 172) (#2531, Cell Signaling), anti-AMPK (#2532, Cell Signaling), Mitophagy Regulators, PINK1 (#6946, Cell Signaling), Parkin (#4211, Cell Signaling), Stress Response: anti-Nrf2 (#12721, Cell Signaling), anti-TFAM (#8076, Cell Signaling) anti-PGC1-alpha (#2178, Cell Signaling). After washing, membranes were incubated with IRDye secondary antibodies for 1 h at room temperature (800CW Goat anti-Rabbit [1:10,000; LicorBio, # 926-32211; Ex/Em: 778/795 nm] or 680RD Goat anti-Mouse [1:10,000; LicorBio, # 926-68070, Ex/Em: 679/696]). Fluorescent signals were visualized using the Odyssey CLx Imager (Licor) imaging system with appropriate infrared fluorescence channels. Band intensity was quantified using Image J software.

### LAMP1/cathepsin B immunostaining

Cells were seeded at a density of 1 × 10^5^ cells per well onto 18-mm glass coverslips (Mercedes Scientific, #R0018) placed in 12-well plates and allowed to adhere for 48 h. Cells were fixed with 4% paraformaldehyde, permeabilized with 0.3% Triton X-100, and blocked with 2% BSA to reduce nonspecific antibody binding. Primary antibodies against LAMP1 (Cell Signaling Technology, #15665) and Cathepsin B (Santa Cruz Biotechnology, #sc-19385) were diluted in 2% BSA and incubated overnight at 4 °C. Cells were then washed with PBS and incubated for 1 h at room temperature with goat anti-mouse IgG (H + L) Alexa Fluor 488 (Thermo Fisher Scientific, #A32723TR) and goat anti-rabbit IgG (H + L) Alexa Fluor 555 (Thermo Fisher Scientific, #A-21428). Coverslips were mounted onto glass slides using Fluoromount Aqueous Mounting Medium (Sigma, #F4680). Images were acquired using a Zeiss LSM 880 confocal microscope equipped with a 63 × /1.46 NA glycerol-immersion objective. Alexa Fluor 488 and Alexa Fluor 555 were excited at 488 nm and 561 nm, respectively. Images were acquired as 2D optical sections under identical acquisition settings across conditions. Colocalization between LAMP1 and Cathepsin B was quantified in Fiji/ImageJ using the BIOP-JACoP plugin, following the approach described by Bolte and Cordelières (99). Pearson’s correlation coefficient and Manders’ overlap coefficients were used to quantify colocalization.

### Mitochondrial structure

To assess mitochondrial morphology, cells were seeded at a density of 1 × 10^5^ cells per well onto 18-mm glass coverslips (thickness 0.16–0.19 mm; Mercedes Scientific #R0018)) placed in 12-well plates and allowed to adhere for 48 h. On the day of imaging, cells were incubated with 150 nM MitoTracker CMXRos (Thermo Fisher Scientific, #M7512) in serum-free DMEM for 30 min at 37 °C to label metabolically active mitochondria. Following staining, cells were washed three times with Hanks’ Balanced Salt Solution (HBSS) to remove unbound dye and maintained in HBSS supplemented with 10% FBS during imaging. Live-cell imaging was performed using a Zeiss LSM 880 confocal microscope equipped with a 100 × /1.46 NA glycerol immersion objective and an environmental chamber maintained at 37 °C with 5% CO_2_. MitoTracker fluorescence was captured using excitation at 579 nm and emission at 599 nm. Z-stack images were acquired to allow 3D reconstruction of mitochondrial networks. Acquired image stacks were analyzed using Imaris software, following the segmentation approach described by Taguchi *et al.* (2021). Mitochondria were identified and segmented based on individual organelle volume and sphericity. For classification, mitochondrial structures were grouped into three distinct morphological categories: fragmented mitochondria were defined by a sphericity value greater than 0.88, intermediate forms had sphericity values between 0.77 and 0.88, and elongated mitochondria displayed sphericity values below 0.77. The proportion of each category was determined by calculating the total mitochondrial volume in each group relative to the total mitochondrial volume per cell.

### Lysosomal pH

Cells were prepared under the same conditions as described for mitochondrial structure analysis. After 48 h of adhesion on 18-mm glass coverslips in 12-well plates, cells were incubated with 100 nM LysoTracker Red DND-99 (Thermo Fisher Scientific, #L7528) and 150 nM of LysoSensor Green DND-189 (Thermo Fisher Scientific, #L7535) in complete medium at 37 °C for 30 min. LysoTracker was used as an acidotropic probe that accumulates in acidic compartments, whereas LysoSensor provides a complementary pH-sensitive fluorescence readout. Following staining, cells were washed three times with phosphate-buffered saline (PBS) to remove excess dye and then incubated in fresh complete medium for an additional 30-min chase period to ensure selective retention of the probe in acidic lysosomal compartments. Live-cell imaging was performed on a Zeiss LSM 880 confocal microscope, and LysoTracker fluorescence was recorded using excitation at 577 nm and emission at 590 nm. LysoSensor fluorescence was acquired under identical imaging conditions across experimental groups using the appropriate excitation and emission settings. High-resolution 2D images were captured under identical acquisition parameters across all samples to allow for comparative quantification. Individual cells were manually segmented using Fiji (ImageJ) software, and mean fluorescence intensity was measured per cell after background fluorescence subtraction. Lysosomal fluorescence intensity was used as a proxy for lysosomal acidification, with lower fluorescence indicating elevated lysosomal pH.

### Seahorse XF metabolic flux analysis

We used the Seahorse XF Cell Mito Stress Test, as we have previously described (31). PLKO and ML1-KD MDA-MB-231 and MCF10A cells were seeded at equal density in Seahorse XF96 cell culture microplates at 5 × 10^4^ cells per well. On the day of the assay, culture medium was replaced with Seahorse XF DMEM medium, pH 7.4 (Agilent, #103575-100), supplemented with 1 mM pyruvate, 2 mM glutamine, and 10 mM glucose. Cells were equilibrated for at least 2 h at 37 °C in a CO_2_-free incubator before analysis. OCR and ECAR were recorded in real time using a Seahorse XFe96 Analyzer and the Agilent Seahorse XF Cell Mito Stress Test Kit (Agilent, #103015-100). Oligomycin, FCCP, and rotenone/antimycin A were sequentially injected at final well concentrations of 2 μM, 2 μM, and 0.5 μM each, respectively. Wells containing assay medium without cells were used as blanks for background correction. OCR and ECAR values were background-corrected using blank wells and analyzed using Seahorse Wave software version 2.6.3. To assess the relative balance between mitochondrial oxidative phosphorylation and glycolytic acidification, OCR/ECAR ratios were calculated under basal and maximal respiratory conditions and for the ATP-linked respiratory component. Results were plotted and statistically analyzed using GraphPad Prism 10.

### Intracellular ATP measurement

Cellular ATP levels were quantified using the Luminescent ATP Detection Assay Kit (Abcam, ab113849) following the manufacturer’s instructions. PLKO control and ML1 knockdown cells were seeded in white-walled, flat-bottom 96-well plates (5000 cells/well, triplicates) and cultured for 48 h at 37 °C with 5% CO_2_. At the time of the assay, plates and reagents were equilibrated to room temperature, and media were gently aspirated. Cells were lysed by adding 50 μl of lysis buffer (Detergent) per well and incubated for 5 min on a shaker. Meanwhile, the substrate solution was prepared by reconstituting the lyophilized enzyme mix (Substrate) in substrate buffer. Immediately after lysis, 50 μl of the reconstituted substrate solution was added to each well. Plates were covered to protect from light and incubated at room temperature for 15 min to allow luminescence stabilization. Luminescence was measured using a Synergy H1 microplate reader (BioTek) with a 1-s integration time. An ATP standard curve (10 μM to 0.01 nM) was generated using serial dilutions of the supplied ATP standard. ATP levels were normalized to cell number determined from parallel wells using manual cell counting.

### Flow cytometry analysis

All flow cytometry assays were performed on a BD FACS Celesta cytometer (BD Biosciences) equipped with a 488-nm laser. A minimum of 20,000 events per sample were collected, and data were analyzed using FlowJo software (v10.7.1). Cells were gated based on forward and side scatter to exclude debris and doublets. *Apoptosis* was assessed using Annexin V (BioLegend, #640906) and 7-Aminoactinomycin D (7-AAD) (BioLegend, #420404) staining. Cells were harvested 72 h post-treatment, washed with Annexin binding buffer (BioLegend, #422201), and incubated with 12.5 μg/ml Annexin V and 20 μg/ml 7-AAD in the dark for 15 min at RT. Fluorescence of Annexin V in PLKO and ML1-KD cells was measured by FITC channel, analyzed by FlowJo software and presented as %. *Cell cycle* distribution was determined using propidium iodide (PI) staining. Cells were synchronized by serum starvation for 12 h, then released into complete medium for 10 h to resume cell cycle progression (31, 100). Following harvesting and washing, cells were fixed in ice-cold 70% ethanol overnight at 4 °C. On the day of analysis, cells were washed with PBS and stained with 100 μg/ml PI and 50 μg/ml RNase A (Sigma) in PBS for 1 h at RT in the dark. DNA content was analyzed in PI channel, analysed by FlowJo software, and presented as %. *Total ROS* levels were measured using oxidation-sensitive dye CMH_2_DCFDA (Thermo Fisher Scientific, #D399). Cells were incubated with 5 μM CMH_2_DCFDA in serum free DMEM for 60 min at 37 °C, then washed twice with PBS and resuspended in 1 ml PBS for acquisition. Mean fluorescence intensity was measured in the FITC channel. *Mitochondrial ROS* was quantified using MitoSOX Red (Thermo Fisher Scientific, #M36008). Cells were incubated with 5 μM MitoSOX in serum free DMEM for 30 min at 37 °C in the dark. After washing, cells were analyzed immediately, and red fluorescence was measured in the PE channel. *Cellular Bioenergetics* (*CENCAT*) was analyzed through non-canonical amino acid tagging as previously described (35, 101, 102). Briefly, cells were cultured in complete DMEM (10% FBS, 1% Anti-Anti) and incubated for 24 h at 37 °C in CO2 incubator. To deplete intracellular methionine, cells were incubated for 1 h at 37 °C in methionine free medium, after which metabolic inhibitors were added at final concentrations of 2 deoxy D glucose (2 DG; 100 μM), oligomycin A (1 μM), or vehicle control, including a combined 2 DG + oligomycin condition. After 15 min, L homopropargylglycine (b HPG; 100 μM) was added without washing, and cells were incubated for an additional 30 min (total inhibitor exposure 45 min). Reactions were stopped by adding a 2 × volume of ice-cold PBS, followed by two washes (500*g*, 5 min). After fixation and permeabilization, incorporated b HPG was detected using a copper catalyzed azide–alkyne cycloaddition reaction (CuSO_4_, ascorbic acid, THPTA ligand, and azide conjugated fluorophore) for 30 min at room temperature. Samples were washed and acquired on a flow cytometer, collecting ≥10,000 live singlet events per sample. Data were analyzed by sequential gating on singlets and b HPG incorporation was quantified as geometric mean fluorescence intensity (GeoMFI). Metabolic dependencies were calculated using four inhibitor conditions—control (Co), 2 DG (DG), oligomycin (O), and combined 2 DG + oligomycin (DGO)—with DGO defining minimal background, using the following formulas: Mitochondrial (OXPHOS) dependence = 100 × (Co − O)/(Co − DGO); and FAO/AAO capacity = 100 × (DG − DGO)/(Co − DGO). A no b HPG control was included to assess nonspecific binding, and assay performance was monitored by ensuring low DGO signal and robust dynamic range.

### Autophagosome–lysosome fusion assay

Cells were seeded on 18-mm glass coverslips in 12-well plates and transfected with the tandem fluorescent reporter plasmid RFP-GFP-LC3 (Addgene #21074) using Lipofectamine 2000 (Thermo Fisher Scientific) according to the manufacturer’s protocol. Twenty-four hours post-transfection, cells were washed with PBS and fixed in 4% paraformaldehyde for 15 min at RT, then mounted using ProLong Diamond Antifade Mountant (Thermo Fisher, #P36961). Fluorescence imaging was performed using a Zeiss LSM 880 confocal microscope equipped with a 63 × oil immersion objective. GFP and RFP were sequentially excited at 488 nm and 561 nm, respectively, and emissions were collected at 509 nm (GFP) and 583 nm (RFP). 2D images were acquired using identical acquisition settings across all samples. Images were analyzed using Fiji (ImageJ). RFP^+^/GFP^+^ (yellow) puncta were counted as autophagosomes, while RFP^+^/GFP^−^ (red-only) puncta were counted as autolysosomes. Quantification was performed manually on a minimum of 20 cells per condition, per experiment, and results were plotted as the ratio of yellow to red puncta (42).

### Mt-mKeima mitophagy assay

Mitophagy was assessed using the pH-sensitive mitochondrial reporter mt-mKeima (103). A stable MDA-MB-231 cell line expressing pHAGE-mt-mKeima (Addgene #131626; provided generously by Dr Cheng) was first generated, after which TRPML1 knockdown was performed as described above to obtain PLKO, ML1-KD1, and ML1-KD3 mt-mKeima-expressing cells. For imaging, cells were seeded at a density of 1 × 10^5^ cells per well onto 18-mm glass coverslips (Mercedes Scientific, #R0018) placed in 12-well plates and allowed to adhere for 48 h. On the day of imaging, culture medium was replaced with phenol-red-free DMEM (Thermo Fisher Scientific, #21063029) supplemented with 10% FBS. Live-cell imaging was performed using a Zeiss LSM 880 confocal microscope equipped with a 63 × /1.46 NA glycerol-immersion objective and an environmental chamber maintained at 37 °C with 5% CO_2_. mt-mKeima emission was collected at 610 to 690 nm following sequential excitation at 458 nm and 561 nm, corresponding to mitochondria in neutral/high-pH and acidic lysosomal compartments, respectively. Three-dimensional Z-stacks were acquired for each field. For quantification, Z-stacks were converted into maximum intensity projections using Zen software. Individual cells were manually segmented in Fiji/ImageJ, and integrated density was measured for both excitation channels after background subtraction. The 561/458 fluorescence ratio was calculated for each cell and used as an index of mitophagy.

### TFEB nuclear translocation assay

Cells were seeded on 18-mm glass coverslips and transfected with GFP-NI-TFEB plasmid (Addgene #38119) using Lipofectamine 2000 (Thermo Fisher Scientific). Twenty-four hours post-transfection, cells were fixed with 4% paraformaldehyde for 15 min at RT before imaging. Imaging was performed using a Zeiss LSM 880 confocal microscope with a 40 × oil immersion objective. GFP was excited at 488 nm and emission was collected at 509 nm. The number of cells with nuclear TFEB was counted and plotted as % of TFEB nucleus positive cells/total cells in the field. At least 10 fields, each 10 to 15 cells, per condition, per experiments, were analyzed and compared across PLKO, ML1-KD, and ML-SA1–treated and untreated PLKO cells.

### Three-dimensional organelle contact analysis

To assess organelle contact remodeling, cells were seeded on 18-mm glass coverslips and transfected with Mito-GFP (for mitochondria), LC3-mCherry (for lysosomes), or Sec61-NEON (for Endoplasmic Reticulum) plasmids. After 24 h, cells were stained with MitoTracker CMXRos (for mitochondria) or LysoTracker Red DND-99 (for lysosomes), depending on the studied organellar contacts. After staining, cells were washed three times with HBSS and maintained in HBSS supplemented with 10% FBS during live-cell imaging. Confocal Z-stacks were acquired using a Zeiss LSM-880 microscope equipped with a 100 × oil immersion objective and an environmental chamber (37 °C, 5% CO_2_). Z-step size was set 0.3 μm to ensure Nyquist sampling for volumetric reconstruction. Fluorophores were imaged using sequential acquisition with fixed laser power and detector settings across all samples. We used the Airyscan detector to achieve higher resolution and sensitivity. This allowed us to visualize cellular organelles more clearly and in greater detail compared to conventional confocal imaging (104). Image analysis was performed using Imaris 10.2 software. Surfaces were generated for mitochondria, lysosomes, and ER compartments using the “Surfaces” module, with intensity thresholds set based on automated background subtraction followed by manual adjustment to capture organelle boundaries. For colocalization of organelles, surface-surface extension in Imaris software was employed. Briefly, “Surface–Surface Contact Area” tool was applied to calculate the contact volume between organelles within a maximum 3D contact distance of 0.2 μm. This distance was chosen to reflect proximity consistent with potential functional tethering (105, 106). Contact parameters were normalized to the total mitochondrial or lysosomal surface volume per cell to account for differences in cell size. A minimum of 10 cells per condition, per experiment, were analyzed. Data were reported as contact volume (μm^3^) or surface area (μm^2^) per cell and expressed as relative fold-change *versus* PLKO controls.

### Mitochondrial and Cytosolic Ca2+ imaging

To measure both mitochondrial and cytosolic Ca^2+^, 50,000 cells were seeded onto 25 mm glass coverslips (Chemglass #CLS-1760) in complete media and allowed to adhere overnight. Coverslips were then mounted into Attofluor cell chambers (Thermo Fisher Scientific #A7816) and cells were suspended in complete media, to which Ca^2+^ sensing dyes were added. For cytosolic Ca^2+^ measurements, the mounted coverslips were incubated with 2 μM Fura-2 AM (Thermo Fisher Scientific #F1225) for 30 minutes at room temperature. Cells were then washed four times with HEPES Buffered Saline Solution (HBSS; 140 mM NaCl, 4.7 mM KCl, 1.13 mM MgCl_2_, 2.0 mM CaCl_2_, 10 mM glucose, and 10 mM HEPES, pH adjusted to 7.4 with NaOH). The chambers were subsequently mounted on an Olympus IX71 Fluorescence Microscope. Cells were imaged with a 20X Fluor objective, and the Fura-2 was excited at 340 and 380 nm using a CoolLED pE-340^fura^ shutter, with emissions captured at a wavelength of 510 nm with a Hamamatsu ORCA-Spark camera. The ratio of the fluorescence at 340 and 380 nm (F_340_/F_380_) was analyzed with HCImage software (https://www.hcimage.com) by drawing regions of interest around the perimeter of approximately 30 cells per coverslip. For mitochondrial Ca^2+^ measurements, the mounted coverslips were incubated with 100 nM MitoTracker Green FM (Thermo Fisher Scientific #M7514) and 1 μM Rhod-2 AM (Thermo Fisher Scientific R1244) for 30 minutes at room temperature. Cells were then washed four times with HBSS and mounted on a Leica Stellaris 5 confocal microscope. Cells were imaged with an HC PL APO CS2 20x/0.75 DRY objective, and MitoTracker Green fluorescence was excited at 490 nm with emissions captured at 516 nm, while Rhod-2 AM was excited at 552 nm with emissions captured at 581 nm. Using the Leica Application Suite X (LAS X) (https://www.leica-microsystems.com), regions of interest were drawn around the mitochondria of approximately 30 cells per coverslip. MitoTracker and Rhod-2 fluorescence were normalized to the fluorescence at t = 0, and the Rhod-2 signal was ratioed against the MitoTracker signal to correct for any changes in focus and eliminate any background cytosolic Rhod-2 signal.

### Metabolomics analysis

Global metabolomics was performed to quantify steady-state intracellular metabolites in control PLKO and ML1-KD MDA-MB-231 and MCF10A cells. For each condition, cells from multiple 35-mm culture dishes were pooled to generate a single biological sample for metabolomic analysis. Cells were scraped in cold (−20 °C) 80% methanol and centrifuged at 13,000*g* for 5 min. A 25 μl aliquot of the resulting supernatant was added to 225 μl of hydrophilic interaction liquid chromatography (HILIC) loading buffer containing 95% acetonitrile, 2 mM ammonium hydroxide, and 2 mM ammonium acetate. After centrifugation at 13,000×*g* for 5 min, triplicate 50 μl injections of each sample were loaded onto an Acquity UPLC BEH Amide column (1.7 μm, 2.1 × 100 mm; Waters #186004801). Targeted metabolite detection was carried out by multiple reaction monitoring (MRM) using a Sciex 5500 QTRAP mass spectrometer, with parameters and acquisition settings as previously described (Almasi *et al.*, 2020). Peak heights for individual metabolites were extracted using Skyline software (MacCoss Lab Software). Significantly altered metabolites were defined as those showing ≥1.5-fold change between KD and PLKO conditions. Pathway enrichment analysis was performed to identify functional metabolic clusters using publicly available metabolic pathway databases. Data were presented as fold change relative to PLKO, and statistical comparisons were performed using Student’s *t* test on at least three independent biological replicates. Because one pooled biological sample was analyzed per condition, the metabolomics data were interpreted descriptively rather than using technical injections as independent biological replicates.

### Statistics

Statistical analyses were conducted using GraphPad Prism 9.0.1 (GraphPad). Unless otherwise specified, Data are presented as means ± SD of at least three biologically independent experiments. For comparisons between two groups, an unpaired Student’s *t* test was used. For comparisons involving more than two groups, one-way or two-way ANOVA followed by Tukey’s multiple-comparison test was used, as appropriate. A *p*-value of less than 0.05 was considered statistically significant. Figure legends specify the statistical tests used for each experiment. The metabolomics analysis was based on one pooled biological sample per condition analyzed by technical replicate injections; these technical replicates were not considered independent biological replicates and were interpreted descriptively.

## Additional Information:

**Table:** 

Antibodies	Catalogue #, Suppliers
anti-Cyclin B	#4138, Cell Signaling
anti-p27	#2552, Cell Signaling
anti-E2F1	#3742, Cell Signaling
anti-Cleaved-Caspase7	#8438, Cell Signaling
anti-Caspase7	#12827, Cell Signaling
anti-Cleaved-Caspase3	#9664, Cell Signaling
anti-Caspase3	#14220, Cell Signaling
anti-p-Bcl-2 (Ser 70)	#2827, Cell Signaling
anti-p21	ab188224, Abcam
anti-EndoG	#4969, Cell Signaling
anti-AIF	#4642, Cell Signaling
anti-MFN1	#14739, Cell Signaling
anti-MFN2	#9482, Cell Signaling
anti-OPA1	#80471, Cell Signaling
anti-p-DRP1 (S616)	#63940, Cell Signaling
anti-p-DRP1 (S637)	#20990, Cell Signaling
anti-MFF	#84580, Cell Signaling
anti-p-P70S6K (Thr 389)	#9206, Cell Signaling
anti-P70S6K	#9202, Cell Signaling
anti-p-ULK1 (S757)	#6888, Cell Signaling
anti-ULK1	#8054, Cell Signaling
anti-p-ulk1 (S555)	#5869, Cell Signaling
anti-LC3	#12741, Cell Signaling
anti-p-AMPK	#5831, Cell Signaling
anti-AMPK	#50081, Cell Signaling
PINK1	#6946, Cell Signaling
anti-Nrf2	#12721, Cell Signaling
anti-Rabbit	IRDye 800CW, 926-32211, Licor
anti-Mouse	IRDye 680RD, 926-68070, Licor
Experimental Models: Cell Lines	
MDA-MB-231	ATTC, Generously Given by Dr. Gujar–Dalhousie University
HCC1806	ATTC, Generously Given by Dr. Gujar–Dalhousie University
HS578T	ATTC, Generously Given by Dr. Gujar–Dalhousie University
MCF-10A	ATTC, Generously Given by Dr. Gujar–Dalhousie University
HEK293T	ATCC, Generously Given by Dr. Linsdell–Dalhousie University
Recombinant DNA	
shTRPML1_1 (ML1-KD1)	Dharmacon, RHS3979-201908411 Sequencing Primer: 5ʹ-AAACCCAGGGCTGCCTTGGAAAAG - 3ʹ
shTRPML1_3 (ML1-KD3)	Dharmacon, RHS3979-201915961 Sequencing Primer: 5ʹ-AAACCCAGGGCTGCCTTGGAAAAG-3ʹ
Lamp1-GFP	Plasmid #16290, Addgene
RFP-GFP-LC3 (pft:LC3)	Plasmid #21074, Addgene, Generously Given by Dr. Dong–Dalhousie University
GFP-NI-TFEB	Plasmid #38119, Addgene, Generously Given by Dr. Pulinilkunnil–Dalhousie University
pHAGE-mt-mKeima	Plasmid #131626; Addgene, Generously Given by by Dr. Cheng–Dalhousie University
Reagents	
Bafilomycin A	Cayman #11038
ML-SA1	Tocris # 47416
ML-SI1	Enzo Life # BML-CR112-0010GW-405833
Doxorubicin	Sigma # 44583
Paclitaxel	Sigma #T7402

## Data availability

All data needed to evaluate the conclusions in the paper are present in the paper. Additional data related to this paper may be requested from the authors.

## Supporting information

This article contains supporting information.

## Conflict of interest

The authors declare the following financial interests/personal relationships which may be considered as potential competing interests:

All authors declare that they have no competing interests. MT is a scientific advisor for Seeker Biologics (Cambridge, MA), Eldec Pharmaceuticals (Durham, NC) and Vivreon Biosciences (San Diego, CA).

## References

[1] Tang T., Yang Z.Y., Wang D., Yang X.Y., Wang J., Li L. (2020). The role of lysosomes in cancer development and progression. Cell Biosci..

[2] Vyas S., Zaganjor E., Haigis M.C. (2016). Mitochondria and cancer. Cell.

[3] Duchen M.R. (2000). Mitochondria and calcium: from cell signalling to cell death. J. Physiol..

[4] Zorov D.B., Juhaszova M., Sollott S.J. (2014). Mitochondrial reactive oxygen species (ROS) and ROS-induced ROS release. Physiol. Rev..

[5] Osellame L.D., Blacker T.S., Duchen M.R. (2012). Cellular and molecular mechanisms of mitochondrial function. Best Pract. Res. Clin. Endocrinol. Metab..

[6] Martinez-Reyes I., Chandel N.S. (2020). Mitochondrial TCA cycle metabolites control physiology and disease. Nat. Commun..

[7] Sivanand S., Viney I., Wellen K.E. (2018). Spatiotemporal control of Acetyl-CoA metabolism in chromatin regulation. Trends Biochem. Sci..

[8] Machado E.R., Annunziata I., van de Vlekkert D., Grosveld G.C., d'Azzo A. (2021). Lysosomes and cancer progression: a malignant liaison. Front. Cell Dev. Biol..

[9] Kirkegaard T., Jaattela M. (2009). Lysosomal involvement in cell death and cancer. Biochim. Biophys. Acta.

[10] Inpanathan S., Botelho R. (2019). The lysosome signaling platform: adapting with the times. Front. Cell Dev. Biol..

[11] Perera R.M., Zoncu R. (2016). The lysosome as a regulatory hub. Annu. Rev. Cell Dev. Biol..

[12] Acharya A., Demetriades C. (2024). mTORC1 activity licenses its own release from the lysosomal surface. Mol. Cell.

[13] Carroll B., Dunlop E.A. (2017). The lysosome: a crucial hub for AMPK and mTORC1 signalling. Biochem. J..

[14] Bartel K., Pein H., Popper B., Schmitt S., Janaki-Raman S., Schulze A. (2019). Connecting lysosomes and mitochondria–a novel role for lipid metabolism in cancer cell death. Cell Commun. Signal..

[15] Martinez-Carreres L., Nasrallah A., Fajas L. (2017). Cancer: linking powerhouses to suicidal bags. Front. Oncol..

[16] Park K., Lim H., Kim J., Hwang Y., Lee Y.S., Bae S.H. (2022). Lysosomal Ca(2+)-mediated TFEB activation modulates mitophagy and functional adaptation of pancreatic beta-cells to metabolic stress. Nat. Commun..

[17] Nezich C.L., Wang C., Fogel A.I., Youle R.J. (2015). MiT/TFE transcription factors are activated during mitophagy downstream of Parkin and Atg5. J. Cell Biol..

[18] Calabrese C., Nolte H., Pitman M.R., Ganesan R., Lampe P., Laboy R. (2024). Mitochondrial translocation of TFEB regulates complex I and inflammation. EMBO Rep..

[19] Xia M., Zhang C., Chen Y., Zhao X., Zhang S., Liu Y. (2022). Transcription factor EB coordinates environmental cues to regulate T regulatory cells' mitochondrial fitness and function. Proc. Natl. Acad. Sci. U. S. A..

[20] Yambire K.F., Rostosky C., Watanabe T., Pacheu-Grau D., Torres-Odio S., Sanchez-Guerrero A. (2019). Impaired lysosomal acidification triggers iron deficiency and inflammation in vivo. Elife.

[21] Peng W., Wong Y.C., Krainc D. (2020). Mitochondria-lysosome contacts regulate mitochondrial Ca(2+) dynamics via lysosomal TRPML1. Proc. Natl. Acad. Sci. U. S. A..

[22] Lee S.H., Duron H.E., Chaudhuri D. (2023). Beyond the TCA cycle: new insights into mitochondrial calcium regulation of oxidative phosphorylation. Biochem. Soc. Trans..

[23] Demers-Lamarche J., Guillebaud G., Tlili M., Todkar K., Belanger N., Grondin M. (2016). Loss of mitochondrial function impairs lysosomes. J. Biol. Chem..

[24] Joshi A.U., Ebert A.E., Haileselassie B., Mochly-Rosen D. (2019). Drp1/Fis1-mediated mitochondrial fragmentation leads to lysosomal dysfunction in cardiac models of Huntington's disease. J. Mol. Cell Cardiol..

[25] Bartel K., Pein H., Popper B., Schmitt S., Janaki-Raman S., Schulze A. (2019). Connecting lysosomes and mitochondria - a novel role for lipid metabolism in cancer cell death. Cell Commun. Signal..

[26] Genovese I., Fornetti E., Ruocco G. (2022). Mitochondria inter-organelle relationships in cancer protein aggregation. Front. Cell Dev. Biol..

[27] Xu M., Almasi S., Yang Y., Yan C., Sterea A.M., Syeda A.K.R. (2019). The lysosomal TRPML1 channel regulates triple negative breast cancer development by promoting mTORC1 and purinergic signaling pathways. Cell Calcium.

[28] Jung J., Cho K.J., Naji A.K., Clemons K.N., Wong C.O., Villanueva M. (2019). HRAS-driven cancer cells are vulnerable to TRPML1 inhibition. EMBO Rep..

[29] Jonsson G., Staaf J., Vallon-Christersson J., Ringner M., Holm K., Hegardt C. (2010). Genomic subtypes of breast cancer identified by array-comparative genomic hybridization display distinct molecular and clinical characteristics. Breast Cancer Res..

[30] Clarke C., Madden S.F., Doolan P., Aherne S.T., Joyce H., O'Driscoll L. (2013). Correlating transcriptional networks to breast cancer survival: a large-scale coexpression analysis. Carcinogenesis.

[31] Gomes G.M.A., Xu M., Syeda A.K.R., Raudonis R., Almasi S., Vijayan V.V. (2025). Targeting TRPML3 inhibits proliferation and invasion, and enhances doxorubicin sensitivity by disrupting lysosomal acidification and mitochondrial function in triple-negative breast cancer. Biochim. Biophys. Acta Mol. Cell Res..

[32] Cheng X., Shen D., Samie M., Xu H. (2010). Mucolipins: Intracellular TRPML1-3 channels. FEBS Lett..

[33] La Thangue N.B. (1994). DP and E2F proteins: components of a heterodimeric transcription factor implicated in cell cycle control. Curr. Opin. Cell Biol..

[34] Sinha K., Das J., Pal P.B., Sil P.C. (2013). Oxidative stress: the mitochondria-dependent and mitochondria-independent pathways of apoptosis. Arch. Toxicol..

[35] Vrieling F., van der Zande H.J.P., Naus B., Smeehuijzen L., van Heck J.I.P., Ignacio B.J. (2024). CENCAT enables immunometabolic profiling by measuring protein synthesis via bioorthogonal noncanonical amino acid tagging. Cell Rep. Methods.

[36] Vargas J.N.S., Hamasaki M., Kawabata T., Youle R.J., Yoshimori T. (2023). The mechanisms and roles of selective autophagy in mammals. Nat. Rev. Mol. Cell Biol..

[37] He C., Klionsky D.J. (2009). Regulation mechanisms and signaling pathways of autophagy. Annu. Rev. Genet..

[38] Yu L., Chen Y., Tooze S.A. (2018). Autophagy pathway: cellular and molecular mechanisms. Autophagy.

[39] Mack H.I., Zheng B., Asara J.M., Thomas S.M. (2012). AMPK-dependent phosphorylation of ULK1 regulates ATG9 localization. Autophagy.

[40] Holczer M., Hajdu B., Lorincz T., Szarka A., Banhegyi G., Kapuy O. (2020). Fine-tuning of AMPK-ULK1-mTORC1 regulatory triangle is crucial for autophagy oscillation. Sci. Rep..

[41] Jiang P., Mizushima N. (2015). LC3- and p62-based biochemical methods for the analysis of autophagy progression in mammalian cells. Methods.

[42] Kaizuka T., Morishita H., Hama Y., Tsukamoto S., Matsui T., Toyota Y. (2016). An autophagic flux probe that releases an internal control. Mol. Cell.

[43] Katayama H., Yamamoto A., Mizushima N., Yoshimori T., Miyawaki A. (2008). GFP-like proteins stably accumulate in lysosomes. Cell Struct. Funct..

[44] Wang Y., Liu N., Huang X., Hu W., Ma Y., Liang Y. (2021). Atmospheric particulate matter impedes autophagic flux by impairing lysosomal milieu and integrity in human umbilical vein endothelial cells (HUVECs). Sci. Total Environ..

[45] Zhang X., Chen Q., Chen M., Ren X., Wang X., Qian J. (2017). Ambroxol enhances anti-cancer effect of microtubule-stabilizing drug to lung carcinoma through blocking autophagic flux in lysosome-dependent way. Am. J. Cancer Res..

[46] Zhao Y., Li Z.F., Zhang D., Wang Z.Y., Wang L. (2021). Quercetin alleviates Cadmium-induced autophagy inhibition via TFEB-dependent lysosomal restoration in primary proximal tubular cells. Ecotoxicol. Environ. Saf..

[47] Wang X.Y., Yang H., Wang M.G., Yang D.B., Wang Z.Y., Wang L. (2017). Trehalose protects against cadmium-induced cytotoxicity in primary rat proximal tubular cells via inhibiting apoptosis and restoring autophagic flux. Cell Death Dis..

[48] Baudot A.D., Haller M., Mrschtik M., Tait S.W., Ryan K.M. (2015). Using enhanced-mitophagy to measure autophagic flux. Methods.

[49] Chu C.T. (2019). Mechanisms of selective autophagy and mitophagy: implications for neurodegenerative diseases. Neurobiol. Dis..

[50] Sun N., Malide D., Liu J., Rovira I.I., Combs C.A., Finkel T. (2017). A fluorescence-based imaging method to measure in vitro and in vivo mitophagy using mt-Keima. Nat. Protoc..

[51] Chen X., Li M., Chen D., Gao W., Guan J.L., Komatsu M. (2012). Autophagy induced by calcium phosphate precipitates involves endoplasmic reticulum membranes in autophagosome biogenesis. PLoS One.

[52] Cheng M., Liu L., Lao Y., Liao W., Liao M., Luo X. (2016). MicroRNA-181a suppresses parkin-mediated mitophagy and sensitizes neuroblastoma cells to mitochondrial uncoupler-induced apoptosis. Oncotarget.

[53] Ding W.X., Yin X.M. (2012). Mitophagy: mechanisms, pathophysiological roles, and analysis. Biol. Chem..

[54] Onishi M., Yamano K., Sato M., Matsuda N., Okamoto K. (2021). Molecular mechanisms and physiological functions of mitophagy. EMBO J..

[55] Marinkovic M., Novak I. (2021). A brief overview of BNIP3L/NIX receptor-mediated mitophagy. FEBS Open Bio.

[56] Wang S., Chen Y., Li X., Zhang W., Liu Z., Wu M. (2020). Emerging role of transcription factor EB in mitochondrial quality control. Biomed. Pharmacother..

[57] Tan S., Yu C.Y., Sim Z.W., Low Z.S., Lee B., See F. (2019). Pomegranate activates TFEB to promote autophagy-lysosomal fitness and mitophagy. Sci. Rep..

[58] Medina D.L., Di Paola S., Peluso I., Armani A., De Stefani D., Venditti R. (2015). Lysosomal calcium signalling regulates autophagy through calcineurin and TFEB. Nat. Cell Biol..

[59] Zhu S.Y., Yao R.Q., Li Y.X., Zhao P.Y., Ren C., Du X.H. (2021). The role and regulatory mechanism of transcription factor EB in health and diseases. Front. Cell Dev. Biol..

[60] Halling J.F., Pilegaard H. (2020). PGC-1alpha-mediated regulation of mitochondrial function and physiological implications. Appl. Physiol. Nutr. Metab..

[61] Campbell C.T., Kolesar J.E., Kaufman B.A. (2012). Mitochondrial transcription factor a regulates mitochondrial transcription initiation, DNA packaging, and genome copy number. Biochim. Biophys. Acta.

[62] Esteras N., Abramov A.Y. (2022). Nrf2 as a regulator of mitochondrial function: energy metabolism and beyond. Free Radic. Biol. Med..

[63] Melone M.A.B., Valentino A., Margarucci S., Galderisi U., Giordano A., Peluso G. (2018). The carnitine system and cancer metabolic plasticity. Cell Death Dis..

[64] Nagarajan S.R., Butler L.M., Hoy A.J. (2021). The diversity and breadth of cancer cell fatty acid metabolism. Cancer Metab..

[65] Li R.J., Xu J., Fu C., Zhang J., Zheng Y.G., Jia H. (2016). Regulation of mTORC1 by lysosomal calcium and calmodulin. Elife.

[66] Wang S., Li H., Yuan M., Fan H., Cai Z. (2022). Role of AMPK in autophagy. Front Physiol..

[67] Kim J., Kundu M., Viollet B., Guan K.L. (2011). AMPK and mTOR regulate autophagy through direct phosphorylation of Ulk1. Nat. Cell Biol..

[68] Lee C., Eldridge M.J.G., Gonzalez-Lozano M.A., Bresnahan T., Niday Z., Del Camino D. (2025). DMXL1 promotes recruitment of V1-ATPase to lysosomes upon TRPML1 activation. Nat. Struct. Mol. Biol..

[69] Bhattacharjee A., Abuammar H., Juhasz G. (2024). Lysosomal activity depends on TRPML1-mediated Ca(2+) release coupled to incoming vesicle fusions. J. Biol. Chem..

[70] Clague M.J., Urbe S. (2025). Diverse routes to mitophagy governed by ubiquitylation and mitochondrial import. Trends Cell Biol..

[71] Wang S., Long H., Hou L., Feng B., Ma Z., Wu Y. (2023). The mitophagy pathway and its implications in human diseases. Signal. Transduct. Target. Ther..

[72] Lynch M.R., Tran M.T., Ralto K.M., Zsengeller Z.K., Raman V., Bhasin S.S. (2020). TFEB-driven lysosomal biogenesis is pivotal for PGC1alpha-dependent renal stress resistance. JCI Insight.

[73] Park J.Y., Kim S., Sohn H.Y., Koh Y.H., Jo C. (2019). TFEB activates Nrf2 by repressing its E3 ubiquitin ligase DCAF11 and promoting phosphorylation of p62. Sci. Rep..

[74] Al Ojaimi M., Salah A., El-Hattab A.W. (2022). Mitochondrial fission and fusion: molecular mechanisms, biological functions, and related disorders. Membranes (Basel).

[75] Chen H., Detmer S.A., Ewald A.J., Griffin E.E., Fraser S.E., Chan D.C. (2003). Mitofusins Mfn1 and Mfn2 coordinately regulate mitochondrial fusion and are essential for embryonic development. J. Cell Biol..

[76] Ge Y., Shi X., Boopathy S., McDonald J., Smith A.W., Chao L.H. (2020). Two forms of Opa1 cooperate to complete fusion of the mitochondrial inner-membrane. Elife.

[77] Wong Y.C., Ysselstein D., Krainc D. (2018). Mitochondria-lysosome contacts regulate mitochondrial fission via RAB7 GTP hydrolysis. Nature.

[78] Todkar K., Ilamathi H.S., Germain M. (2017). Mitochondria and lysosomes: discovering bonds. Front. Cell Dev. Biol..

[79] Bayliak M.M., Lushchak V.I. (2021). Pleiotropic effects of alpha-ketoglutarate as a potential anti-ageing agent. Ageing Res. Rev..

[80] Son E.D., Choi G.H., Kim H., Lee B., Chang I.S., Hwang J.S. (2007). Alpha-ketoglutarate stimulates procollagen production in cultured human dermal fibroblasts, and decreases UVB-induced wrinkle formation following topical application on the dorsal skin of hairless mice. Biol. Pharm. Bull..

[81] Cluntun A.A., Lukey M.J., Cerione R.A., Locasale J.W. (2017). Glutamine metabolism in cancer: understanding the heterogeneity. Trends Cancer.

[82] Bernfeld E., Foster D.A. (2019). Glutamine as an essential amino acid for KRas-Driven cancer cells. Trends Endocrinol. Metab..

[83] Diehl F.F., Miettinen T.P., Elbashir R., Nabel C.S., Darnell A.M., Do B.T. (2022). Nucleotide imbalance decouples cell growth from cell proliferation. Nat. Cell Biol..

[84] Awoyomi O.F., Gorospe C.M., Das B., Mishra P., Sharma S., Diachenko O. (2025). RRM2B deficiency causes dATP and dGTP depletion through enhanced degradation and slower synthesis. Proc. Natl. Acad. Sci. U. S. A..

[85] Qu Q., Zeng F., Liu X., Wang Q.J., Deng F. (2016). Fatty acid oxidation and carnitine palmitoyltransferase I: emerging therapeutic targets in cancer. Cell Death Dis..

[86] Ma Y., Temkin S.M., Hawkridge A.M., Guo C., Wang W., Wang X.Y. (2018). Fatty acid oxidation: an emerging facet of metabolic transformation in cancer. Cancer Lett..

[87] Farahzadi R., Hejazi M.S., Molavi O., Pishgahzadeh E., Montazersaheb S., Jafari S. (2023). Clinical significance of carnitine in the treatment of cancer: from traffic to the regulation. Oxid Med. Cell. Longev..

[88] Zhang J., Wu G., Zhu H., Yang F., Yang S., Vuong A.M. (2022). Circulating carnitine levels and breast cancer: a matched retrospective case-control study. Front. Oncol..

[89] Basho R.K., Gilcrease M., Murthy R.K., Helgason T., Karp D.D., Meric-Bernstam F. (2017). Targeting the PI3K/AKT/mTOR pathway for the treatment of mesenchymal triple-negative breast cancer: evidence from a phase 1 trial of mTOR inhibition in combination with liposomal Doxorubicin and Bevacizumab. JAMA Oncol..

[90] Weiner-Gorzel K., Murphy M. (2021). Mitochondrial dynamics, a new therapeutic target for triple negative Breast Cancer. Biochim. Biophys. Acta Rev. Cancer.

[91] Zhang Y., Ji Y., Tu Y., Li Y. (2025). Autophagy in doxorubicin resistance: basic concepts, therapeutic perspectives and clinical translation. Front. Immunol..

[92] Chen G., Wang F., Trachootham D., Huang P. (2010). Preferential killing of cancer cells with mitochondrial dysfunction by natural compounds. Mitochondrion.

[93] Wu H.L., Gong Y., Ji P., Xie Y.F., Jiang Y.Z., Liu G.Y. (2022). Targeting nucleotide metabolism: a promising approach to enhance cancer immunotherapy. J. Hematol. Oncol..

[94] Dong X.P., Cheng X., Mills E., Delling M., Wang F., Kurz T. (2008). The type IV mucolipidosis-associated protein TRPML1 is an endolysosomal iron release channel. Nature.

[95] Feng X., Huang Y., Lu Y., Xiong J., Wong C.O., Yang P. (2014). Drosophila TRPML forms PI(3,5)P2-activated cation channels in both endolysosomes and plasma membrane. J. Biol. Chem..

[96] Kim H.J., Li Q., Tjon-Kon-Sang S., So I., Kiselyov K., Soyombo A.A. (2008). A novel mode of TRPML3 regulation by extracytosolic pH absent in the varitint-waddler phenotype. EMBO J..

[97] Kim S.W., Kim M.K., Hong S., Choi A., Choi J.H., Muallem S. (2022). The intracellular Ca(2+) channel TRPML3 is a PI3P effector that regulates autophagosome biogenesis. Proc. Natl. Acad. Sci. U. S. A..

[98] Klages N., Zufferey R., Trono D. (2000). A stable system for the high-titer production of multiply attenuated lentiviral vectors. Mol. Ther..

[99] Bolte S., Cordelieres F.P. (2006). A guided tour into subcellular colocalization analysis in light microscopy. J. Microsc..

[100] Almasi S., Long C.Y., Sterea A., Clements D.R., Gujar S., El Hiani Y. (2019). TRPM2 silencing causes G2/M arrest and apoptosis in lung cancer cells via increasing intracellular ROS and RNS levels and activating the JNK pathway. Cell Physiol. Biochem..

[101] Dieterich D.C., Link A.J., Graumann J., Tirrell D.A., Schuman E.M. (2006). Selective identification of newly synthesized proteins in mammalian cells using bioorthogonal noncanonical amino acid tagging (BONCAT). Proc. Natl. Acad. Sci. U. S. A..

[102] Ignacio B.J., Dijkstra J., Mora N., Slot E.F.J., van Weijsten M.J., Storkebaum E. (2023). THRONCAT: metabolic labeling of newly synthesized proteins using a bioorthogonal threonine analog. Nat. Commun..

[103] Vargas J.N.S., Wang C., Bunker E., Hao L., Maric D., Schiavo G. (2019). Spatiotemporal control of ULK1 activation by NDP52 and TBK1 during selective autophagy. Mol. Cell.

[104] Wu X., Hammer J.A. (2021). ZEISS Airyscan: optimizing usage for fast, gentle, super-resolution imaging. Methods Mol. Biol..

[105] Valm A.M., Cohen S., Legant W.R., Melunis J., Hershberg U., Wait E. (2017). Applying systems-level spectral imaging and analysis to reveal the organelle interactome. Nature.

[106] Wong Y.C., Kim S., Peng W., Krainc D. (2019). Regulation and function of mitochondria–lysosome membrane contact sites in cellular homeostasis. Trends Cell Biol..

