# Supplementary material for: TRPML1 loss disrupts lysosome–mitochondria homeostasis and sensitizes MDA-MB-231 triple-negative breast cancer cells to chemotherapy
Source: J Biol Chem. 2026 Sep 23;302(11):113596. doi: 10.1016/j.jbc.2026.113596 (PMC13734889; doi:10.1016/j.jbc.2026.113596)
Supplement: Supplementary file 1 [file mmc1.pdf]

TRPML1 signaling at the lysosome–mitochondria nexus sustains mitophagy, metabolic fitness, and chemotherapy tolerance in MDA-MB-231 triple-negative breast cancer cells

By

Alia K. Syeda[1], Shekoufeh Almasi[1], Cory J Benson,[2,3,4,5], Barry E. Kennedy[6], Ryan M. Yoast[2,7], Scott M. Emrich[2,7], Amari Delima-Baril[1] Logan Slade[8,9], Shanmugasundaram Pakkiriswami[8,10], Vishnu V. Vijayan[6], Unnikrishnan B. Sheela[6], Shashi Gujar[6], Thomas Pulinilkunnil[8], Mohamed Trebak[2,3,4,5,#], Yassine El Hiani[1#]

From

[1]Department of Physiology and Biophysics, [6]Microbiology and Immunology of Dalhousie University, 5850 College Street, Halifax, NS, Canada; [8]Department of Biochemistry and Molecular Biology, Dalhousie University, Dalhousie Medicine New Brunswick, Saint John, NB, Canada

[2]Graduate Program in Cellular and Molecular Physiology, The Pennsylvania State University College of Medicine, Hershey, Pennsylvania, USA; [3]Department of Pharmacology and Chemical Biology, [4]Vascular Medicine Institute, and [5]UPMC Hillman Cancer Center. University of Pittsburgh School of Medicine, Pittsburgh, Pennsylvania, United States

[7]Current Address: Senior Scientist, Bristol Myers Squibb, 3551 Lawrenceville Rd, Princeton, NJ 08540

[9]Current Address: Department of Biochemistry and Biophysics, University of North Carolina at Chapel Hill, NC, USA

[10]Current Address: Department of Physiology, University of Minnesota, Minneapolis, MN, USA

[#]Correspondence should be addressed to either Mohamed Trebak (trebakm@pitt.edu) or Yassine El Hiani (yassine.elhiani@Dal.Ca)

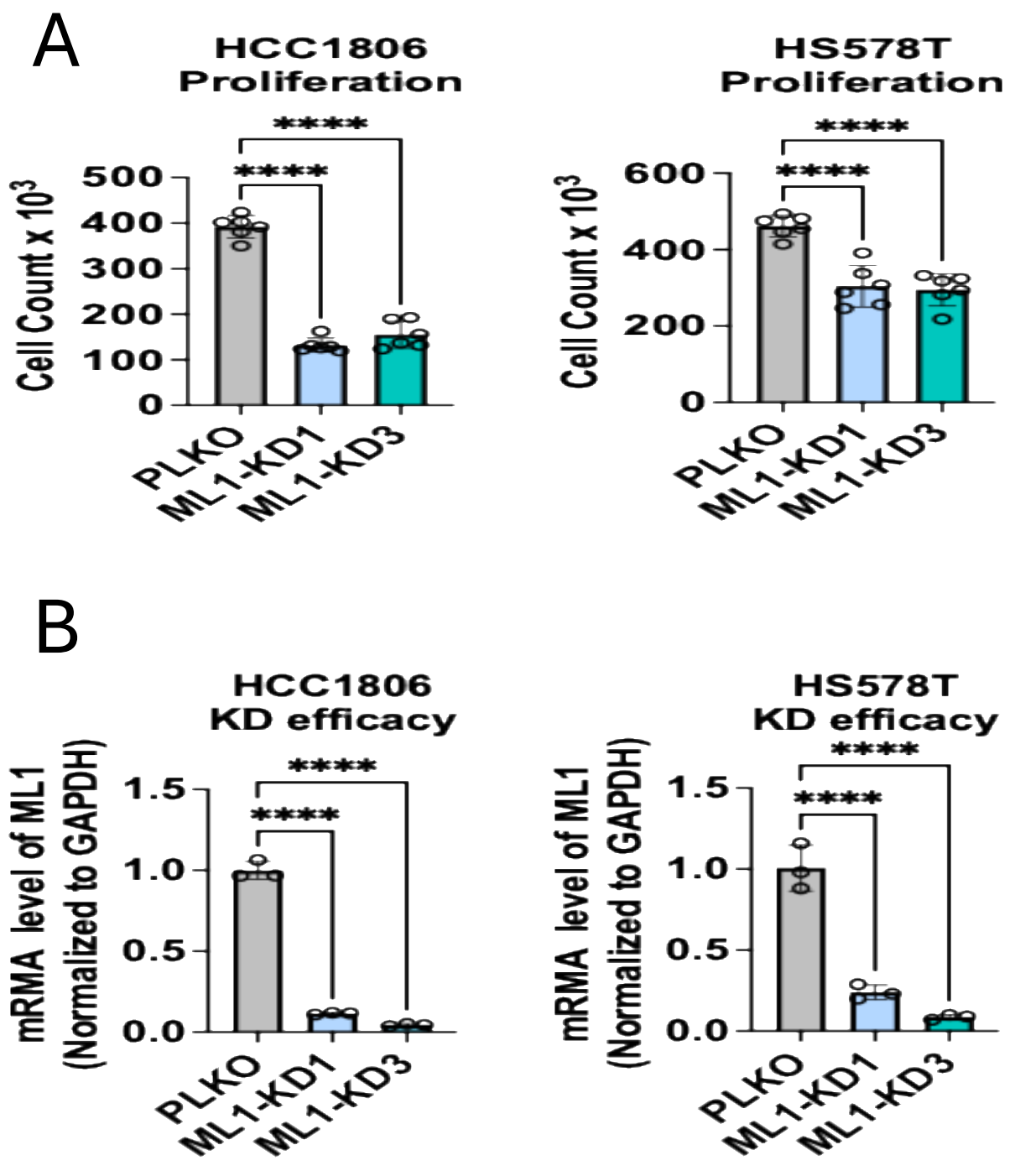

Figure Supplementary 1: TRPML1 knockdown reduces proliferation in HCC1806 and HS578T TNBC cells. **(A)** 1.2 x 10^5 cells were seeded, and cell proliferation was determined by cell counting after 48 h; **(B)** TRPML1 knockdown efficiency in HCC1806 and HS578T cells expressing control PLKO or two independent TRPML1-targeting shRNAs (ML1-KD1 and ML1-KD3) was confirmed by qPCR. Data are presented as mean ± SD. Statistical significance was determined by one-way ANOVA with Tukey's multiple-comparison test. ****$P < 0.0001$.

Fig1-Supp. Syeda et al., 2026

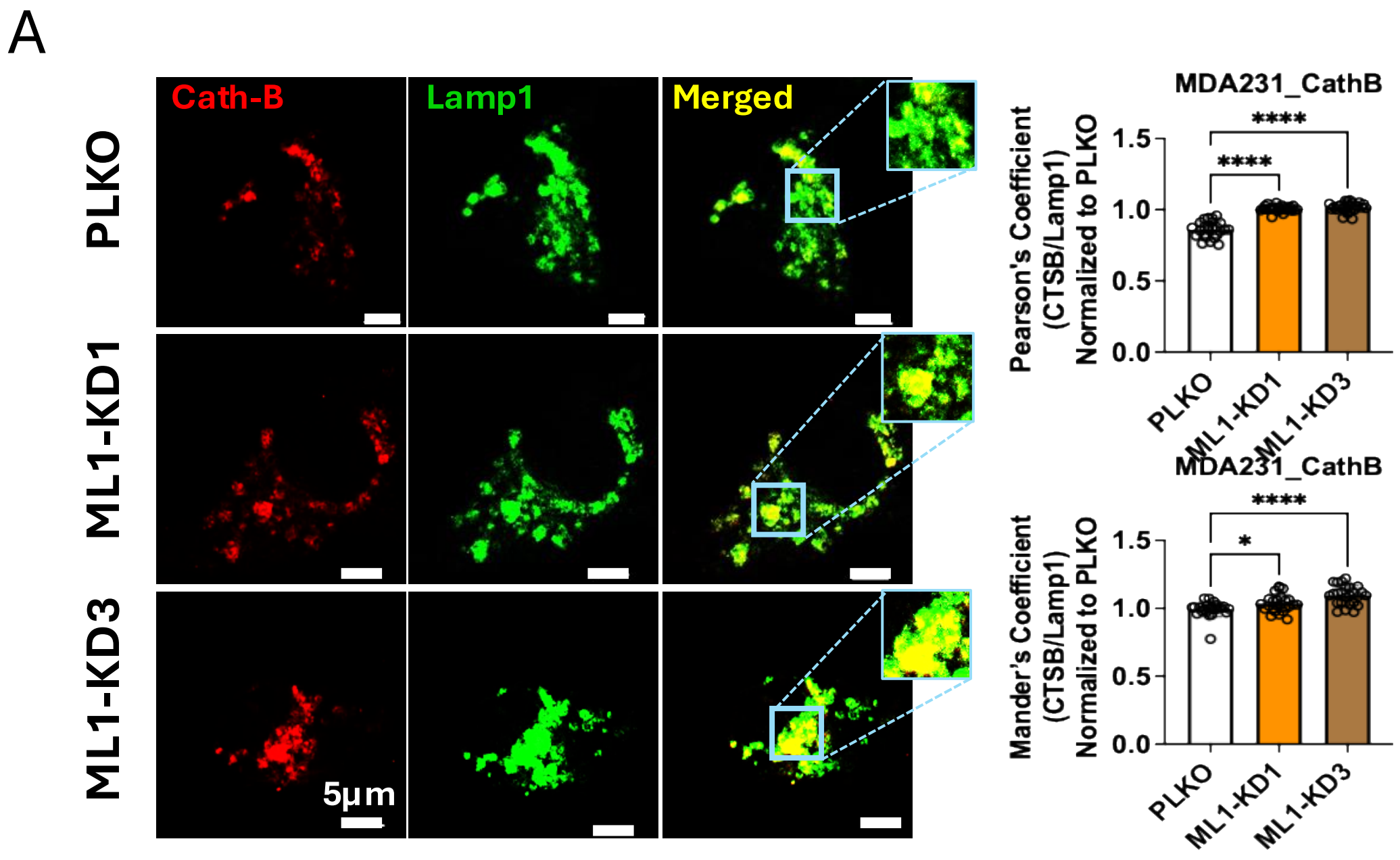

Figure Supplementary 2: TRPML1 knockdown has no effect on Cathepsin B distribution nor Galectin-3 levels in MDA-MB-231 cells. Representative single Z-Stack planes, using confocal live-cell imaging with 100x magnification, from MDA-MB-231 PLKO and ML1-KD cells, stained with Cathepsin B (red signal) and Lamp1 (green signal) cells – Scale bar: 5 µm; Merge panels show the degree of signal overlapping between Cathepsin B and Lamp1. Graph (Means ± SD) represents the Pearson's and Mander's coefficient in PLKO vs ML1-KD cells. Data were obtained from three independent biological experiments, 25 cells per condition. Statistical significance was determined by one-way ANOVA with Tukey's multiple comparison test for PLKO vs ML1-KD1/ML1-KD3) of MDA-MB-231. $^{*}P < 0.05$, and $^{***}P < 0.001$.

Fig2-Supp. Syeda et al., 2026

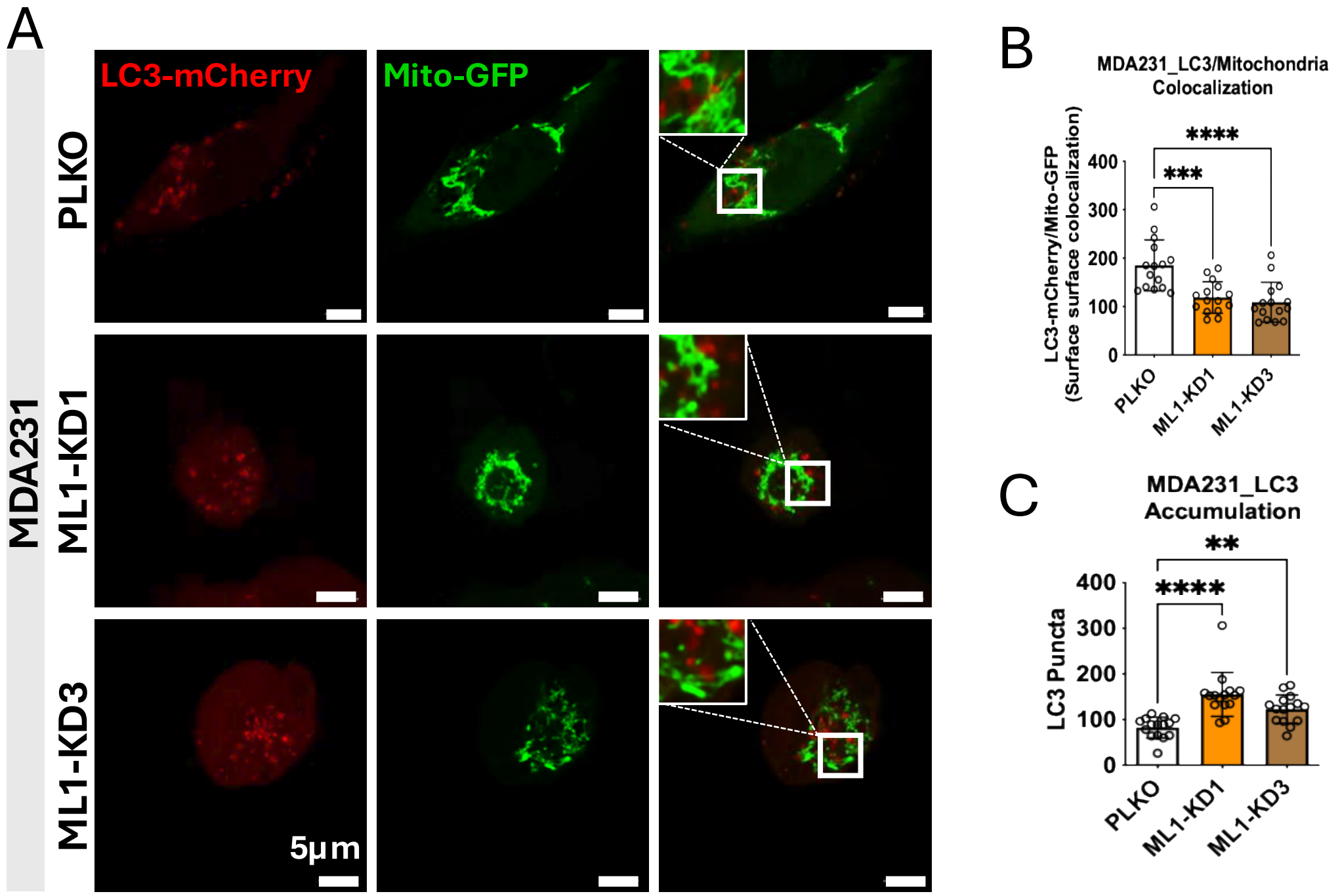

Figure Supplementary 3: TRPML1 knockdown inhibits mitophagy in MDA-MB-231 cells.

(A) Representative single Z-Stack planes, using confocal live-cell imaging with 100x magnification, from MDA-MB-231 PLKO and ML1-KD cells, co-transfected with LC3-mCherry and Mito-GFP to assess mitophagy. Red (LC3) and green (mitochondria) channels, as well as merged images, are shown – Scale bar: 5 µm. Merge panels show the degree of volume overlapping between autolysosomes and mitochondria. (B) Graph (Means ± SD) represents the % of surface-surface colocalization between autolysosomal volume that are shared with mitochondrial volume in PLKO vs ML1-KD cells. (C) LC3-mCherry puncta (autolysosomes) were quantified to evaluate LC3 levels in PLKO vs ML1-KD conditions. In B and C, data were obtained from three independent biological experiments, 15 cells per condition; Statistical significance was determined by one-way ANOVA with Tukey's multiple comparison test for PLKO vs ML1-KD1/ML1-KD3 MDA-MB-231.

Fig3-Supp. Syeda et al., 2026
